# Biotin Transport-Targeting Polysaccharide-Modified PAMAM G3 Dendrimer as System Delivering α-Mangostin into Cancer Cells and *C. elegans* Worms

**DOI:** 10.3390/ijms222312925

**Published:** 2021-11-29

**Authors:** Joanna Markowicz, Łukasz Uram, Stanisław Wołowiec, Wojciech Rode

**Affiliations:** 1 Faculty of Chemistry, Rzeszow University of Technology, 6 Powstancow Warszawy Ave., 35-959 Rzeszow, Poland; luram@prz.edu.pl; 2 Medical College, Rzeszow University, 1a Warzywna Str., 35-310 Rzeszow, Poland; swolowiec@ur.edu.pl; 3 Nencki Institute of Experimental Biology, 3 Pasteur Street, 02-093 Warsaw, Poland

**Keywords:** α-mangostin, poly(amidoamine) dendrimer, targeted drug delivery, biotin targeting, glioblastoma multiforme, squamous cell carcinoma, antiparasitic therapy

## Abstract

The natural xanthone α-mangostin (αM) exhibits a wide range of pharmacological activities, including antineoplastic and anti-nematode properties, but low water solubility and poor selectivity of the drug prevent its potential clinical use. Therefore, the targeted third-generation poly(amidoamine) dendrimer (PAMAM G3) delivery system was proposed, based on hyperbranched polymer showing good solubility, high biocompatibility and low immunogenicity. A multifunctional nanocarrier was prepared by attaching αM to the surface amine groups of dendrimer via amide bond in the ratio 5 (G3^2B12gh5M^) or 17 (G3^2B10gh17M^) residues per one dendrimer molecule. Twelve or ten remaining amine groups were modified by conjugation with D-glucoheptono-1,4-lactone (gh) to block the amine groups, and two biotin (B) residues as targeting moieties. The biological activity of the obtained conjugates was studied in vitro on glioma U-118 MG and squamous cell carcinoma SCC-15 cancer cells compared to normal fibroblasts (BJ), and in vivo on a model organism *Caenorhabditis elegans*. Dendrimer vehicle G3^2B12gh^ at concentrations up to 20 µM showed no anti-proliferative effect against tested cell lines, with a feeble cytotoxicity of the highest concentration seen only with SCC-15 cells. The attachment of αM to the vehicle significantly increased cytotoxic effect of the drug, even by 4- and 25-fold for G3^2B12gh5M^ and G3^2B10gh17M^, respectively. A stronger inhibition of cells viability and influence on other metabolic parameters (proliferation, adhesion, ATP level and Caspase-3/7 activity) was observed for G3^2B10gh17M^ than for G3^2B12gh5M^. Both bioconjugates were internalized efficiently into the cells. Similarly, the attachment of αM to the dendrimer vehicle increased its toxicity for *C. elegans*. Thus, the proposed α-mangostin delivery system allowed the drug to be more effective in the dendrimer-bound as compared to free state against both cultured the cancer cells and model organism, suggesting that this treatment is promising for anticancer as well as anti-nematode chemotherapy.

## 1. Introduction

The isoprenylated xanthone α-mangostin (αM) was firstly isolated from the pericarp of mangosteen tree (*Garcinia mangostana* L., *Clusiaceae*) that has long been used in traditional medicine in Southeast Asia to treat inflammation, ulcer, skin infection, and wounds [1]. Nowadays, numerous in vitro and in vivo studies have proved that mangostins possess diverse pharmacological activities, such as antioxidant, anti-inflammatory, antibacterial, antifungal, antimalarial, anticancer and anthelmintic [2,3,4,5]. 

Considering the potential pharmacological application of αM, it is significantly limited by its low water solubility (2.03 × 10^−4^ mg/L at 25 °C) [6], a major obstacle in achieving maximum drug bioavailability and accumulation in the target organs. The latter, together with insufficient target selectivity in the human body, resulted in the fact that αM has not been approved for clinical use. 

One strategy increasing the concentration of biologically active substances in target locations and cells is to take advantage of a drug delivery agent [7], e.g., to bind a drug with nanoparticles, in order to improve its physicochemical properties and transport. Among the αM delivery systems tested so far, nanolipids, nanopolymers, nanomicelles, nanoliposomes, nanoemulsion, nanofibers and metal nanoparticles have been studied [8], e.g., αM-loaded fibroin nanoparticles (FNPs) crosslinked with EDC or PEI, compared to the free αM, confirmed the crosslinked FNPs to increase the drug’s solubility by about 3-fold [9], as well as to offer sustained drug release and reduced hematotoxicity. Anticancer studies of these nanoparticles with Caco-2 colorectal and MCF-7 breast adenocarcinoma cell lines showed αM to cause apoptosis with cytotoxicity exceeding that of free drug. Additionally, cyclodextrin-based hyperbranched polymer nanoparticles (CDNPs) were used [10] to solubilize αM encapsulated in the CD cavity. They assumed that release of αM in the slow mode is essential for its retention until the cancerous region is reached. Samprasit et al. [11] used αM-loaded mucoadhesive nanoparticles as colon-targeted drug delivery. They proved that chitosan and thiolated chitosan nanoparticles crosslinked by genipin (GP) and modified by Eudragit^®^ L100 increased αM loading limited the release of the drug in the upper gastrointestinal tract, and enhanced its delivery to the colon.

The vast majority of the proposed carriers encapsulate αM; moreover, there are many potential carriers that allow binding it via covalent bonds and significantly increase the stability of such a system. An interesting example presents poly(amidoamine) dendrimers (PAMAM) that are spheroidal or ellipsoidal three-dimensional polymers with the peripheral functional groups and internal cavities. PAMAM dendrimers may be favorably used in drug delivery due to their hydrophilic, biocompatible, and non-immunogenic nature [12], having been applied as targeting drug or gene delivery systems and as diagnostic agents. Drugs or biologically active molecules can be encapsulated in the interior space of dendrimers, attracted by electrostatic interactions or linked via surface groups [13], but covalently attached drugs, e.g., methotrexate, are more stable compared to non-covalent drugs [14]. Additionally, polyvalency of PAMAMs has a potential role in intracellular targeted drug delivery by surface modification [13]. Especially in anticancer therapy, drug targeting is important in order to maximize the therapeutic potential in tumor area with minimizing side effects in normal tissues [15]. The PAMAM dendrimers present the platform for surface modification with numerous ligand moieties that can improve active targeting to the cancer cells and increase tumor specificity with minimum systemic toxicity [12]. Examples of such ligands include monoclonal antibodies, polypeptides, antibody–drug conjugates, nucleic acids and signal transduction inhibitors [16]. Additionally, vitamins, such as folic acid and biotin, are promising molecules targeting nanoparticles and potential drugs conjugates into cancer cells overexpressing folate and biotin receptors, respectively, e.g., breast, ovarian, lung, and colon cancer cells [17,18,19]. Notably, biotinylated dendrimers have been demonstrated to be absorbed into cells stronger than non-biotinylated [20,21].

The aim of this study was to design, synthesize, characterize, and investigate the biological, anti-cancer activity of biotin-targeted, polysaccharide modified PAMAM G3 dendrimers substituted covalently by αM (G3^2B12gh5M^ and G3^2B10gh17M^, differing by the substitution level) against human grade IV glioma U-118 MG cells, human squamous cell carcinoma (SCC-15), compared to normal human fibroblasts (BJ), with the three cell lines found previously to accumulate biotin [22]. The effects of the above-mentioned conjugates or their vehicle (G3^2B12gh^) on viability, proliferation, adhesion, apoptosis, as well as the intracellular ATP level were determined. Cellular accumulation and localization of fluorescently labeled analogues were also evaluated. Additionally, the in vivo toxicity of dendrimer conjugates and αM was tested with model nematode *Caenorhabditis elegans*, which has been used extensively in toxicological studies of many nanoparticles and as a model organism providing insights into cancer cells metabolism, also allowing to assess the anti-parasitic activity.

## 2. Materials and Methods

### 2.1. Reagents

α-Mangostin (αM, purity ≥ 98% (HPLC)) was purchased from Aktin Chemicals, Inc. (Chengdu, China). Ethylenediamine, methyl acrylate, D-glucoheptono-1,4-lactone (GHL), biotin *N*-hydroxysuccinimide ester (NHS-Biotin), 4-nitrophenyl chloroformate (NPCF), 2-Chloro-1-methylpyridinium iodide (Mukaiyama reagent), 4-dimethylaminopyridine (DMAP), 6-[fluorescein-5(6)-carboxamido]hexanoic acid (FCH), dimethyl sulfoxide (dmso) and other reagents used in syntheses were obtained from Merck KGaA (Darmstadt, Germany). Spectra/Por^®^ 3 RC dialysis membrane (cellulose, MW_cutoff_–3.5 kD) was provided by Carl Roth GmbH & Co. KG (Karlsruhe, Germany).

Human glioblastoma (U-118 MG, ATCC^®^ HTB-15), human squamous cell carcinoma (SCC-15, ATCC^®^ CRL-1623), and human normal fibroblast (BJ, ATCC^®^ CRL-2522) cell lines were purchased from the American Type Culture Collection (ATCC, Manassas, VA, USA). Dulbecco’s Modified Eagle’s Media (DMEM and DMEM/ F-12), Eagle’s Minimum Essential Medium (EMEM), and fetal bovine serum (FBS) were obtained from Corning Inc. (New York, NY, USA). Penicillin and streptomycin solution, phosphate-buffered saline (PBS) with and without magnesium and calcium ions, and DAPI (4′,6-diamidino-2-phenylindole, dihydrochloride), Hoechst 33342, and MitoTracker™ Deep Red FM were provided by Thermo Fisher Scientific Inc. (Waltham, MA, USA). Trypsin-EDTA solution, hydrocortisone, 0.33% neutral red solution (3-amino-m-dimethylamino-2-methyl-phenazine hydrochloride), XTT sodium salt (2,3-bis[2-methoxy-4-nitro-5-sulfophenyl]-2H-tetrazolium-5-carboxanilide inner salt), phenazinemethosulfate (PMS), crystal violet, 0.4% trypan blue solution, dimethylsulfoxide (dmso) for molecular biology, 5-Fluoro-2′-deoxy-uridine (FUdR), and other chemicals and buffers were purchased from Merck KGaA (Darmstadt, Germany). CellTiter-Glo^®^ Luminescent Cell Viability Assay and Apo-ONE^®^ Homogenous Caspase-3/7 Assay were obtained from Promega Corporation (Madison, WI, USA). Cell cultures dishes and materials were from Corning Incorporated (Corning, NY, USA), Greiner (Kremsmünster, Austria), or Nunc (Roskilde, Denmark). All reagents used for *C. elegans* culture and synchronization were supplied by Sigma-Aldrich (Saint Louis, MO, USA) or Carl Roth GmbH & Co., KG (Karlsruhe, Germany).

### 2.2. Chemical Syntheses and Purification Methods

#### 2.2.1. PAMAM G3 Conjugation with Biotin

PAMAM G3 dendrimer was synthesized starting from an ethylenediamine core by repeatable two-step procedure according to Tomalia’s protocol [23] and stored as 20.1 mM solution in methanol for further use. Then PAMAM G3, after prior methanol evaporation, was substituted with two equivalents of biotin by stepwise addition of 99 mg (290 μmoles) of solid NHS-Biotin to 695 mg of G3 (145 μmoles) dissolved in 4 mL of dmso with vigorous stirring. The solution was left at 50 °C for 4 h, then transferred into dialytic tube (cellulose, MW_cutoff_ = 3.5 kD) and dialyzed for 3 days against water (7 × 3 L). Afterwards, the solvent was removed by vacuum rotary evaporation and dried under high vacuum overnight. The product was identified by ^1^H NMR spectroscopy as G3 substituted with average two residues of amide-bonded biotin G3^2B^, as described before [24,25]. The isolated yield of the product was 47% (501 mg; 68 μmoles; MW_calc_ = 7362 g mol^−1^).

#### 2.2.2. Activation of α-Mangostin with NPCF and Attachment to G3^2B^

α-Mangostin (αM) was activated with 4-nitrophenyl chloroformate (NPCF; 10% molar excess), which further provided one carbon linker between hydroxyl group of αM and amino group of PAMAM G3. Thus, 223.3 mg (544 μmoles) of αM was dissolved in 25 mL of chloroform, then 120.6 mg (598 μmoles) of NPCF was added in portions as solid with vigorous stirring. To this solution, 146.6 mg (1200 μmoles) of 4-dimethylaminopyridine (DMAP) was added and the mixture was heated under reflux for 4 h. Then, chloroform was removed by vacuum rotary evaporation and remained solid dried under high vacuum. The solid product, α-mangostin 4-nitrophenylcarbonate (αM-NPC) was dissolved in 10 mL dmso to obtain 54.4 mM stock solution. This solution (6 mL, 326.4 μmoles of αM-NPC, 143 mg of αM) was added dropwise into 162 mg of G3^2B^ (22 μmoles) in 1.6 mL dmso to obtain highly substituted conjugate [A]. In another synthesis process, stock solution of αM-NPC (2 mL, 109 μmoles) was added to 22 μmoles of G3^2B^ in 1.6 mL dmso [B]. In both cases, the mixtures were heated at 50 °C for 3 h, then dialyzed and isolated solids were dried in vacuo. Yields: 273.2 mg, 91.4% for [A], identified by ^1^H NMR spectroscopy as G3^2B17M^ and 188.7 mg, 90% for [B], identified by ^1^H NMR spectroscopy as G3^2B5M^. Long-lasting dialysis of synthesized conjugates, especially G3^2B17M^ (4 days, 12 times 5 L of receiving water), resulted in loss of low-substituted fraction of G3^2BxM^ (x < 5). During the dialytic process, immediate precipitation of highly substituted derivatives was observed. The solids inside dialytic tubes were irritated throughout dialysis in order to remove solid-entrapped low molecular weight DMAP, *p*-nitrophenol, dmso, and other reagents. The products [A] and [B] were further converted by reaction with GHL (vide infra).

#### 2.2.3. G3^2B^ and G3^2BM^ Supplementation with D-Glucoheptono-1,4-lactone

To the solutions of G3^2B17M^ and G3^2B5M^ obtained in the previous step according to protocol [A] and [B] (ca 20 μmoles each), in 5 mL dmso, 264 μmoles of solid GHL (54.9 mg) were added in portions with vigorous stirring. The mixtures were left for 16 h at 50 °C. Thereafter, purification was performed according to the procedure described earlier (3 days of dialysis followed by 24 h drying under high vacuum).

**Scheme 1:**
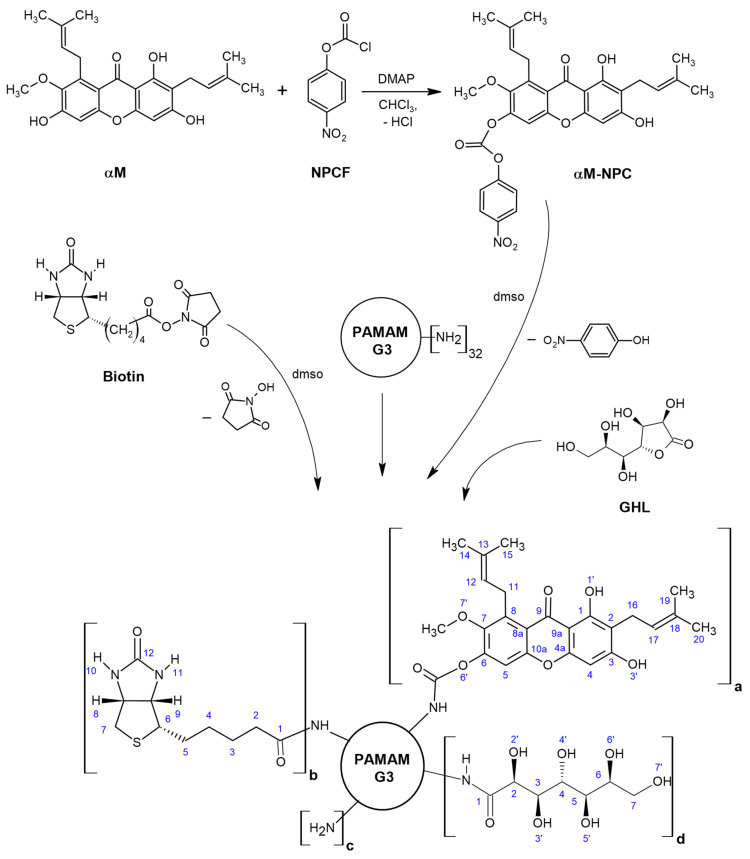
The scheme of synthesis of dendrimer PAMAM G3 conjugates with αmangostin, biotin and D-glucoheptono-1,4-lactone attached via amide bond. The following conjugates were obtained: G3^2B12gh5M^ (a = 5, b = 2, c = 13, d = 12), G3^2B10gh17M^ (a = 17, b = 2, c = 3, d = 10), and G3^2B12gh^ (a = 0, b = 2, c = 18, d = 12). Abbreviations: NPCF—4-nitrophenyl chloroformate, αM-NPC—p-nitrophenyl) carbonate derivative of αM, DMAP—4-dimethylaminopyridine.

The steps of synthesis of dendrimer PAMAM G3 conjugates with α-mangostin, biotin and D-glucoheptono-1,4-lactone is presented in the Scheme 1.

**Figure 1:**
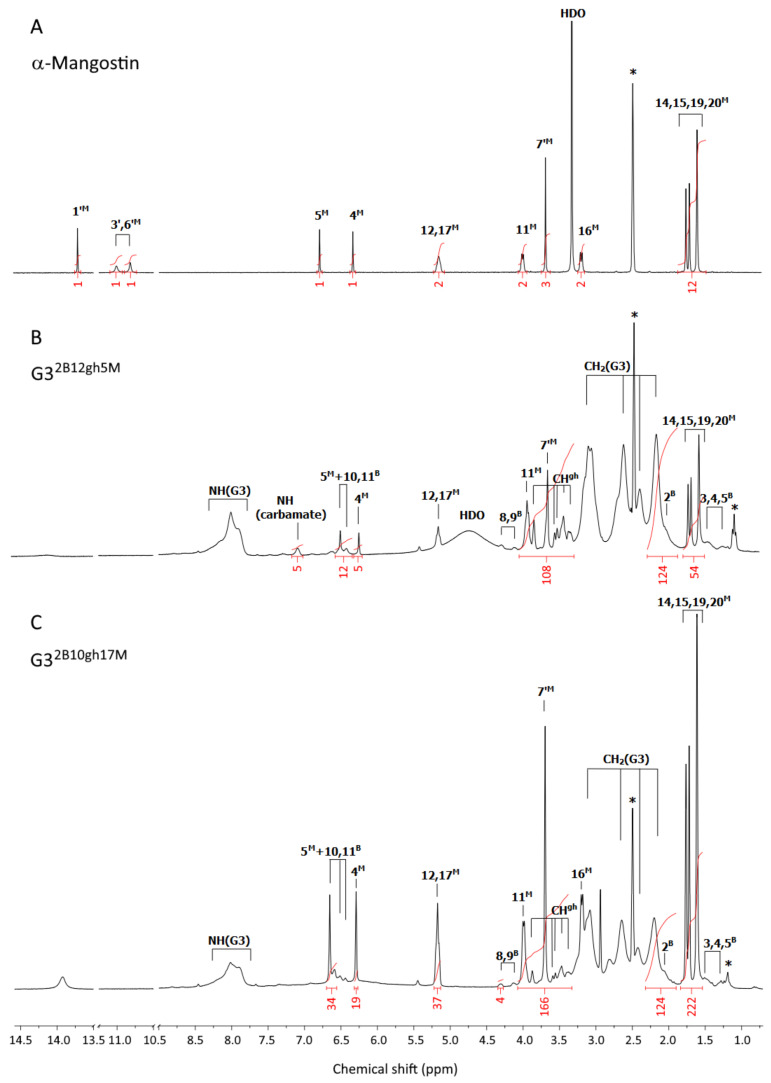
The ^1^H-NMR spectra of α-mangostin (**A**), G3^2B12gh5M^ (**B**), and G3^2B10gh17M^ (**C**) conjugates in dmso-d_6_. The residual solvent peak at 2.5 and impurity resonances are marked with asterisks *. The PAMAM G3 core dendrimer resonances are labeled as CH_2_(G3) and NH(G3) in spectrum B and C. The resonances of α-mangostin, biotin, and glucoheptoamide are labeled by locants with ^M^, ^B^, and ^gh^ upper indexes, respectively, according to the atom numbering in Scheme 1.

**Figure 2:**
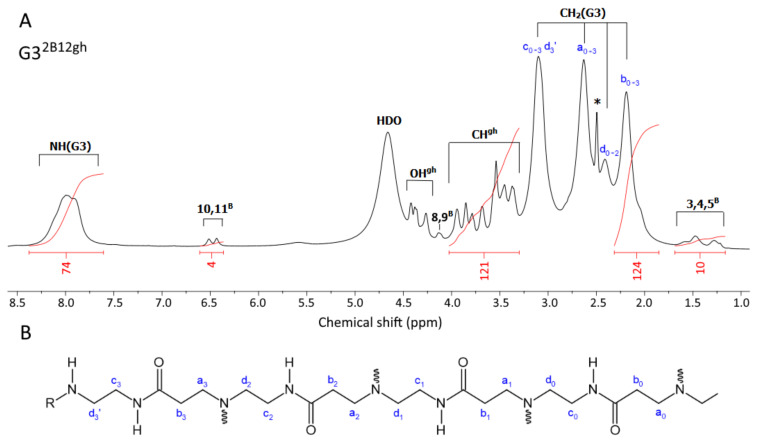
(**A**) The ^1^H-NMR spectrum of G3^2B12gh^ vehicle in dmso-d_6_. The PAMAM G3 core dendrimer resonances are labeled as CH_2_(G3) and NH(G3). The resonances of biotin and D-glucoheptoamide residues are labeled with locants with ^B^ and ^gh^. The residual solvent peak is labeled with an asterisk *. For atom numbering, see Scheme 1. (**B**) The selected chain of the PAMAM G3 dendrimer core with carbon atom numbering. Branch points at ternary nitrogen atoms are marked with wavy lines. R = H, biotin or gh residues.

The obtained conjugates were characterized by the ^1^H NMR (Figure 1 and Figure 2), and 2-D ^1^H-^1^H COSY spectroscopy in dmso-d_6_ (Figure S1), which revealed the level of PAMAM G3^2B^ substitution by αM and gh residues: G3^2B12gh^, G3^2B12gh5M^, and G3^2B10gh17M^, with the isolated yield 94.5% (20.8 μmoles, MW_calc_ = 9859 g mol^−1^), 86% (19 μmoles, MW_calc_ = 11,660 g mol^−1^), and 91% (20 μmoles, MW_calc_ = 16,896 g mol^−1^), respectively.

#### Analytical Data

^1^H NMR (dmso-d_6_; for atom numbering see Scheme 1): chemical shift [ppm] (intensity, multiplicity, assignment):

α-Mangostin (Figure 1A): 13.72 ([1H], s, 1′^M^); 11.01 ([1H], bs, 3′^M^); 10.82 ([1H], bs, 6′^M^); 6.80 ([1H], s, 5^M^); 6.34 ([1H], s, 4^M^); 5.16 ([2H], t overlapped, 12,17^M^); 4.01 ([2H], d, 11^M^); 3.70 ([3H], s, 7′^M^); 3.34 (s, HDO); 3.21 ([2H], d, 16^M^); 1.70 ([12H], m, 14,15,19, 20^M^).

G3^2B12gh5M^ (Figure 1B): 7.10 ([5H], s, NH(carbamate)); 6.51 ([5H], s, 5^M^); 6.26 ([5H], s, 4^M^); 5.17 ([10H], t overlapped, 12,17^M^); 3.95 ([10H], m, 11^M^); 3.68 ([15H], s, 7′^M^); 1.69 ([60H], overlapped s, 14,15,19,20^M^); PAMAM G3 CH_2_ broad resonances: 3.12, 2.64, 2.42, 2.19 [484H]; PAMAM G3 NH: 8.0 ppm, [73H]; Biotin resonances: 6.43 ([4H], bs, 10^B^ and 11^B^); 4.31 and 4.14 ([2H] and [2H], 8^B^ and 9^B^); 2.04 ([4H], 2^B^); 1.49–1.22 ([12H], 3^B^,4^B^,5^B^); gh resonances: 3.87 and 3.58–3.36 ([84H], s and m, CH^gh^).

G3^2B10gh17M^ (Fig 1C): 6.65 ([17H], s, 5^M^); 6.29 ([17H], s, 4^M^); 5.16 ([34H], t overlapped, 12,17^M^); 3.99 ([34H]; d, 11^M^); 3.70 ([51H], s, 7′^M^); 3.19 ([34H], d, 16^M^); 1.70 ([204H], four overlapped singlets, 14,15,19,20^M^); PAMAM G3 CH_2_ broad resonances: 3.14–2.20 [484H]; PAMAM G3 NH: 8.02 ppm, [60H]; biotin resonances: 6.51 and 6.44 ([4H], s and s, 10^B^ and 11^B^); 4.31 and 4.13 ([4H], s and s, 8^B^ and 9^B^); 2.06 ([4H], 2^B^); 1.51–1.28 ([12H], 3^B^,4^B^,5^B^); gh resonances: 3.87 and 3.59–3.37 ([70H], s and m, CH^gh^).

G3^2B12gh^ (Figure 2A): 6.47 ([4H], d, 10,11^B^); 4.36 (overlapped singlets, OH^gh^); 3.66 ([84H], m, CH^gh^); 1.39 ([12H], m, 3,4,5^B^); PAMAM G3 CH_2_ broad resonances: 3.10, 2.63, 2.41, 2.19 [480H]; PAMAM G3 NH broad resonance is centered at 8.00 ppm, [78H].

#### 2.2.4. Fluorescent Labeling of the Conjugates

Fluorescently labeled analogues of G3^2B12gh5M^ and G3^2B10gh17M^ were synthesized by attachment of one equivalent of 6-[fluorescein-5(6)-carboxamido]hexanoic acid (FCH) via an ester bond. The carboxyl groups of FCH were activated by Mukaiyama reagent (2-Chloro-1-methylpyridinium iodide). Specifically, 9.0 mg (18 μmoles) of solid FCH was dissolved in 1 mL of dmso with vigorous stirring and protection from light. Then, 6.2 mg (27 μmoles) of Mukaiyama reagent and 6.6 mg (54 μmoles) of DMAP were added with stirring and the mixture was heated at 45 °C for 0.5 h. The activated FCH was divided into two parts and added dropwise to 0.8 mL (7.1 μmoles) of G3^2B12gh5M^ and 2 mL (7.8 μmoles) of G3^2B10gh17M^ in dmso in equimolar amount. Both reaction mixtures were kept on the heating block at 45 °C overnight followed by purification as before. The ^1^H NMR spectra in dmso-d_6_ were taken and confirmed the attachment of one equivalent of FCH to the dendrimer conjugates. The isolated yield for G3^2B12gh5MF^ was 59% (4.19 μmoles, MW_calc_ = 12,150 g mol^−1^) and for G3^2B10gh17MF^ was 53% (4.1 μmoles, MW_calc_ = 17,386 g mol^−1^). These fluorescent-labeled compounds were used for cellular internalization studies by CLSM.

### 2.3. NMR Spectroscopy

The 1D ^1^H and ^13^C NMR spectra and 2D ^1^H-^1^H correlations spectroscopy (COSY), ^1^H-^13^C heteronuclear single quantum correlation (HSQC), and heteronuclear multiple bond correlation (HMBC) spectra were recorded in dmso-d_6_ using Bruker 300 MHz instrument (Rheinstetten, Germany) at College of Natural Sciences, University of Rzeszów.

### 2.4. Conjugate Size and ζ Potential Measurements

Size and ζ potential of G3^2B12gh^, G3^2B12gh5M^, and G3^2B10gh17M^ conjugates were measured using the dynamic light scattering technique at pH 5 (0.05 M acetate buffer) and in water using the Zetasizer Nano instrument (Malvern, UK) for 1 mg/mL samples (0.7–1.0 mM solutions).

### 2.5. Biological Studies

#### 2.5.1. Cell Cultures

Human glioblastoma cells (U-118 MG, doubling time—37 h) were grown in DMEM, human squamous carcinoma cells (SCC-15, doubling time—48 h) were cultured in DMEM F-12 supplemented with hydrocortisone (400 ng/mL), and normal human skin fibroblasts (BJ, doubling time—1.9 day) were cultured in EMEM. Culture media were supplemented with heat-inactivated 10% FBS and 100 U/mL penicillin and 1% streptomycin solution. All cell lines were cultured at 37 °C in a humidified atmosphere of 95% air with 5% CO_2_. Growth media were changed every 2–3 days and cells were passaged at 80–85% confluence with 0.25% trypsin-0.03% EDTA in PBS (calcium and magnesium ions free). Cell morphology was observed with Nikon TE2000S Inverted Microscope (Tokyo, Japan) with phase contrast. Viability and cell density were estimated by trypan blue exclusion test using Automatic Cell Counter TC20TM (BioRad Laboratories, Hercules, CA, USA). All assays were performed in triplicates in three independent experiments. The working solutions of synthesized dendrimer conjugates were prepared from stock solutions in a corresponding cell culture media by adjusting the dmso concentration to 0.05–0.2% (depending on the type of conjugate), which had no effect on treated cells. Control samples with non-treated cells in complete culture medium with adjusted dmso concentration were included in all assays.

#### 2.5.2. Cytotoxicity (NR and XTT Assays)

The cytotoxicity of PAMAM G3 conjugates with αM (G3^2B12gh5M^ or G3^2B10gh17M^) and the G3^2B12gh^ vehicle was assessed with neutral red uptake (NR) assay and XTT reduction assay. Cells were seeded in flat, clear bottom 96-well culture plates in triplicate (100 μL cell suspension/well) at a density of 1 × 10^4^ cells/well and allowed to attach for 24 h. Then, cells were incubated with working solutions of dendrimer conjugates and vehicle in complete culture medium for 48 h. After that, the NR assay and XTT reduction assay were performed as described in Uram et al. [20]. Additionally, to present cells morphology and the level of accumulation of neutral red dye in lysosomes, microscopic images were collected with a Delta Optical IB-100 microscope with contrast phase under 200× magnification.

#### 2.5.3. Fluorescently Labeled G3^2B12gh5M^ or G3^2B10gh17M^ Cellular Accumulation and Distribution

Cellular accumulation as well as colocalization with nuclei and mitochondria of fluorescently labeled dendrimer conjugates were analyzed using confocal microscopy. Cells were seeded into an 8-well Lab-tek™ Chambered Coverglass (Nunc, Denmark) with a borosilicate glass bottom at a density of 7 × 10^4^ cells/well in 400 μL and placed in an incubator for 24 h. Then, BJ, U118 MG or SCC-15 cells were incubated with non-toxic concentrations of FCH-labeled G3^2B12gh5M^ (1 μM concentration) and G3^2B10gh17M^ (0.1 μM concentration) conjugates, respectively, for 4 or 48 h. After incubation, the dendrimer solution was replaced with an 8 μM Hoechst 33,342 and 50 nM MitoTracker Deep Red FM mixture in growth medium without FBS and incubated at 37 °C for 15 min. Following nuclei and mitochondria staining, cells were washed with PBS (three times) and fixed with 3.7% formaldehyde in PBS. The images were collected in three channels with a confocal microscope (Olympus Fluoview FV10i, Tokyo, Japan) at 361/497 nm for Hoechst, 491/515 nm for FCH, and 644/665 nm for MitoTracker. Images were collected using an objective with water immersion, under a total magnification of 240×. The obtained images had an optical section thickness of app. 1.2 μm. Image analysis was performed with the ImageJ software.

#### 2.5.4. Apoptosis and Intracellular ATP Level

The activity of caspase-3 and -7, the apoptosis marker, was measured to check the ability of the synthesized compounds to induce apoptosis in studied cells. In addition, the intracellular ATP level was assessed. For both parameters, commercially available kits Apo-ONE^®^ Homogenous Caspase-3/7 Assay (Promega) and CellTiter-Glo^®^ Luminescent Cell Viability Assay (Promega) were used. Cells were plated at a density of 1 × 10^4^ per well into a flat, black bottom 96-microplates for apoptosis assay and into a clear 96-microplate for ATP level assay. After 24 h of incubation, cells were treated with the G3^2B12gh5M^ or G3^2B10gh17M^ solutions (100 μL/well) for a further 48 h. Both assays were carried out as described by Uram et al. [26], who used Hoechst 33342 staining to determine the number of cells.

#### 2.5.5. Proliferation

Cell proliferation was estimated using DAPI staining. The 5 × 10^3^ cells/well were seeded into a flat, clear bottom 96-well plates and incubated for 24 h at 37 °C to attach. After removing the growth media, cells were treated with working solutions of G3^2B12gh5M^ or G3^2B10gh17M^ conjugates for 72 h in increasing concentrations. In the next step, plates were centrifuged (5 min, 700 g) and the medium was gently removed. The assay was performed according to Uram et al. [26].

#### 2.5.6. Adhesion

Evaluation of cell adhesion was performed with crystal violet (CV) assay. Cells were seeded into a 96-well clear bottom plates at a density of 1 × 10^4^ cells/well and left 24 h at 37 °C to attach. Afterwards, cells were incubated with working solutions of studied compounds for 48 h and the subsequent operations were carried out as described in Markowicz et al. [5]. Images of the studied cells stained with crystal violet were collected with a Delta Optical IB-100 microscope with contrast phase, with a 10× objective magnification.

#### 2.5.7. Toxicity to Caenorhabditis Elegans and the Worm Survival Analysis

The model organism *Caenorhabditis elegans* was used to estimate in vivo activity of the synthesized dendrimer conjugates and α-mangostin alone in the multicellular system. The wild type *C. elegans* strain N2, variety Bristol, was cultured at 20 °C on NGM agar plates with *E. coli* OP50 lawn as a food source [27]. Survival assay was based on the protocol by Bischof et al. [28] and was adapted to a 96-well plate. Worms at L4 stage were used in the assay and obtained from eggs by prior synchronization of all-stages-nematode population by treatment with hypochlorite. The obtained eggs were left in M9 buffer overnight at room temperature for hatching. The next day, nematodes at L1 stage were transferred to NGM plates with bacterial lawn and grown at 20 °C until the L4 stage (approx. 44 h). After that, the L4 worms were washed twice with distilled water and centrifuged at 250 g followed by resuspension in complete S medium ([29] and centrifugation. Then, the density of nematode suspension was estimated according to Scanlan et al. [30]. Nematodes were suspended in complete S medium supplemented with E. coli OP50 (1:1000), 0.08% cholesterol (5 mg/mL in Et-OH), 1% penicillin-streptomycin, 1% nystatin, and 100 mM FUdR (in final concentration 200 μM) to obtain 20 nematodes in 50 μL transferred to each well. FUdR was added to sterilize nematodes. The working solutions of tested compounds were prepared in the complete S medium with dmso (adjusted to 0.1%, 0.36%, and 0.41% for αM, G3^2B12gh5M^, and G3^2B10gh17M^, respectively) and pipetted (50 μL/well) to a 96-well plate with previously seeded worms. Then, worms were incubated at 20 °C for 7 days. Every day live (moving and curling) and dead nematodes were counted under an inverted microscope. Additionally, images of some morphological changes in nematodes after incubation with G3^2B12gh5M^ were collected using Delta Optical IB-100 microscope under 10× and 20× magnification. The assay was performed in triplicates in three independent experiments.

#### 2.5.8. Statistical Analysis

Due to the lack of a normal distribution of the data in the experimental groups, the non-parametric Kruskal–Wallis test was used to evaluate the differences between dendrimer-treated cells and non-treated control in each cell line. To determine the statistically significant differences between G3^2B12gh5M^ and G3^2B10gh17M^ treated groups, a Mann–Whitney *U* test was performed. *p* ≤ 0.05 was considered statistically significant. The survival curves of *C. elegans* were presented in a plot of the Kaplan–Meier estimator. Statistically significant differences (with *p* ≤ 0.05) between treated and non-treated control groups were indicated using Gehan’s Wilcoxon test. All analyses and calculations were performed using Statistica 13.3 software (StatSoft Poland, Cracow).

## 3. Results and Discussion

### 3.1. Dendrimer Conjugates Synthesis and Characterization

The bioavailability of α-mangostin (αM) is low due to its poor solubility in aqueous solutions, below 0.5 µM at ambient temperature. On the other hand, it has been evidenced that αM shows a wide range of pharmacological activities, including anticancer, antioxidant, anti-inflammatory [31], and antibacterial [32]. In vitro and in vivo studies on various cancer models have proven that the anticancer activity of αM is manifested by, i.a., inhibition of cell proliferation, induction of apoptosis [33,34], and inhibition of metastasis [35]. In order to overcome limitations of αM, we performed several attempts to functionalize it by attachment it to PAMAM dendrimers, which are known to act both as solubilizers for hydrophobic drug molecules and as macromolecular carriers for covalently attached (pro)drugs [36]. Thus, we have used poly(amidoamine) dendrimer of the third generation, G3, as macromolecular carrier, and attempted to bind αM via short link provided by *p*-nitrophenyl chloroformate (NPCF). αM was functionalized with NPCF to obtain *p*-nitrophenylcarbonate derivative, αM-NPC which was further used to bind it to terminal primary amine groups of G3. Although we were able to isolate G3-αM with NPCF-derived carbonyl linker, the water solubility of obtained derivatives was too low to progress with biological tests. Therefore, we converted G3-αM conjugates into more soluble compounds by reaction of remained primary amine groups with D-glucoheptono-1,4-lactone (GHL) to obtain amide-attached polyhydroxyalkyl chains. Two G3-αM derivatives were prepared (Scheme 1) differing by αM substitution levels amounting on average to 17 and 5 residues per dendrimer molecule. These were additionally furnished with two equivalents of amide-attached biotin per dendrimer molecule and exhaustively or semi-exhaustively glucoheptoamidated with GHL. The macromolecular G3 substrate was equipped with 2 biotin to obtain G3^2B^ as before, reacted with αM-NPC, and further converted with excess of GHL, as described before [24]. Additionally, in order to obtain the control carrier, void of αM, G3 substrate was substituted with two biotin residues per dendrimer molecule and further glucoheptoamidated with excess of GHL to obtain G3^2B12gh^. The conjugates were well soluble in dmso and characterized by NMR spectroscopy in this solvent, while molecular size was determined by DLS measurements in diluted aqueous solutions.

The ^1^H NMR spectra of the final products, G3^2B12gh5M^ and G3^2B10gh17M^, are presented in Figure 1B,C. For comparison, the ^1^H-NMR spectrum of αM in dmso-d_6_ was recorded (Figure 1A) and ^1^H resonances were assigned in accordance with literature data [37,38]. Common features of the ^1^H NMR spectra were the PAMAM G3 core methylene proton (a,b,c,d) resonances within 2.19–3.10 ppm region, while protons of amide groups gave broad signal at 8.0 ppm (NH(G3)). In the case of biotin, resonances of aliphatic chain protons (2^B^ and 3,4,5^B^), ureido ring protons (10,11^B^), and thiophane ring protons (8,9^B^) were separated from gh and G3 core resonances (Figure 1B,C). The resonances of gh C-H protons were observed in the 3.3–3.9 ppm region.

For both dendrimer conjugates G3^2B12gh5M^ and G3^2B10gh17M^, the characteristic resonances of αM in the ^1^H NMR spectra are observed (Figure 1): partially overlapped singlets within 1.61–1.77 ppm from protons of four methyl groups, and methoxy group proton singlet resonance at 3.70 ppm. Although prenyl methylene proton (11 and 16) doublets remain non-equivalent in both G3^2B12gh5M^ and G3^2B10gh17M^, the 11-H doublet was found at 3.99 ppm (for G3^2B10gh17M^) and at 3.95 (for G3^2B12gh5M^). The 12 and 17-H triplets are overlapped at 5.16 ppm for αM, G3^2B12gh5M^, and G3^2B10gh17M^.

The resonances of two αM aromatic protons in both conjugated spectra are considerably shifted upfield, due to the shielding effect of the hyperbranched dendrimer with additional polyhydroxyalkylamide chains. The larger shift of 5^M^ resonance in both conjugates related to αM (0.30 ppm upfield) in comparison with the 4^M^ signal (0.08 ppm upfiled) were observed. The ^1^H and ^13^C resonances of αM in G3^2B12gh5M^ and G3^2B10gh17M^ were assigned based on 2-D ^1^H-^1^H COSY (Figure S1) and heteronuclear ^1^H-^13^C HSQC and HMBC experiments (for combined HSQC/HMBC map see Figure S2) and are listed in Table S1.

The considerable shift of H5 proton resonance (0.30 ppm upfield) together with 4.4 ppm downfield shift of C6 and 1.5 ppm upfield shift of C7 resonance in relation to αM alone spectra in dmso-d_6_ strongly suggest that 6′ oxygen is involved in carbamide linker. In such a bonding mode, both prenyl proton multiplets remain unshifted in the conjugates.

We were not able to isolate an αM-NPC intermediate single crystal to verify unambiguously which hydroxyl group was substituted with *para*-nitrophenylcarbonate and further involved in a bonding with dendrimer.

Additionally, G3^2B12gh^ conjugate was synthesized to examine the vehicle cytotoxicity. All of these conjugates were extensively purified by dialysis against water, in order to remove low molecular reagents or side products, such as *p*-nitrophenol and DMAP, and dried under high vacuum. The ^1^H NMR spectrum of G3^2B12gh^ conjugate is shown in Figure 2A. The ^1^H resonances were assigned based on COSY spectrum as described before [24]. The number of attached residues of αM, biotin and gh was estimated based upon the integral intensity of ^1^H resonances in relation to the reference intensity of b_0-3_ protons resonance from the dendrimer core, namely [120H].

Because no free amine groups of G3 were readily available in G3^2B10gh17M^, all conjugates were single-labeled with fluorescein using FCH, which was activated with Mukaiyama reagent and attached to gh hydroxyl groups via ester bond. The average 1:1 stoichiometry was confirmed by ^1^H NMR spectra (not shown) in G3^2B12gh5MF^ and G3^2B10gh17MF^.

### 3.2. Size and Zeta Potential of Conjugates

**Figure 3:**
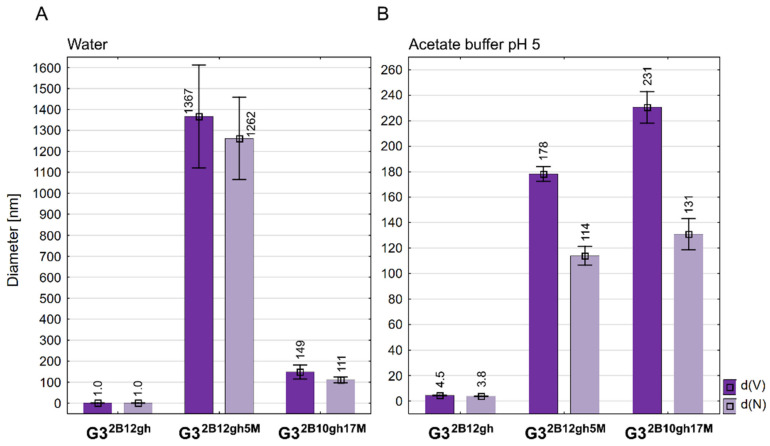
Diameter of conjugates averaged by volume (d(V)) and by number of molecules (d(N)) measured in water (**A**) and in acetate buffer pH 5 (**B**). Data are presented as mean ± standard deviation with mean values marked above the columns.

Unlike PAMAM G3, the G3^2B12gh^ derivative has smaller dynamic diameter in water, which increases upon acidification of the solution to pH 5 about four times (Figure 3; for numbers, see Table 1) due to protonation of internal tertiary amine groups, as observed before within a series of G3^gh^ derivatives [39]. The highest value of diameter was observed already for half-substituted G3^16gh^, namely 4.60 nm (averaged by volume) or 3.80 nm (averaged by number); the biotin-containing conjugate G3^2B12gh^ has a comparable size.

Although we have prepared two relatively well water-soluble αM conjugates, namely G3^2B12gh5M^ and G3^2B10gh17M^, in contrary to non-glucoheptoamidated semiproducts, the size measurements of aqueous solutions evidenced high association of each G3^2B12gh5M^ and G3^2B10gh17M^. DLS measurements indicated the number-averaged size for G3^2B12gh5M^, amounting to 1262 nm with major contribution from large size particles (d(V) = 1367 > d(N)). These associates were destabilized at pH 5, resulting in a decrease of particle size within the <200,100> nm region. The G3^2B10gh17M^ conjugate did not show an additional association in neutral aqueous solution, and finally, the size of particles was within <230,100> nm regardless of the pH (7 or 5). Nonetheless, both conjugates showed major contribution of larger associates in acidic solution, which is illustrated by d(V) > d(N) in Figure 3B.

**Figure 4:**
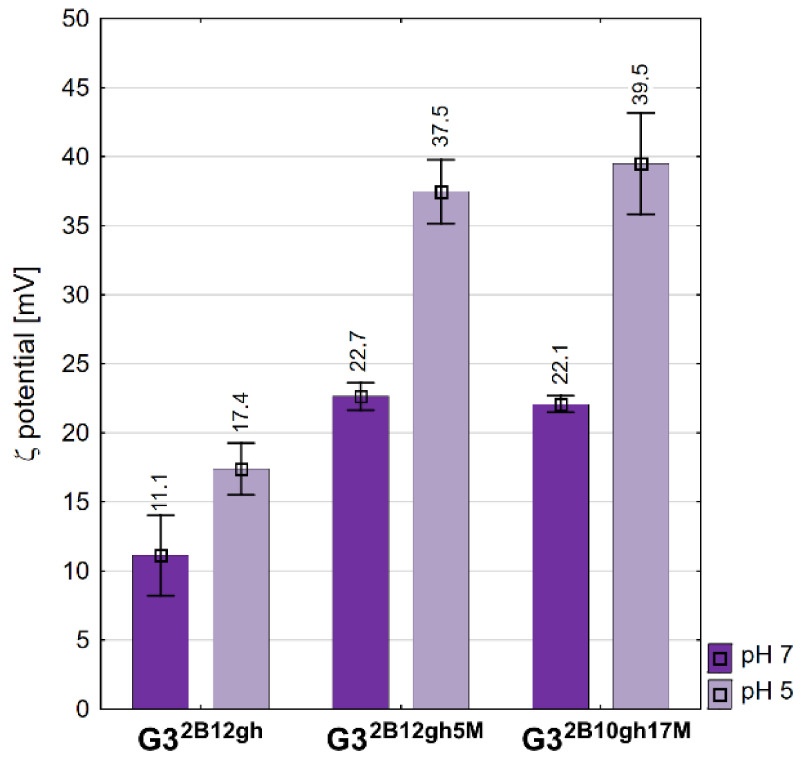
Zeta potential values of conjugates measured in water and acetate buffer pH 5. Data are presented as mean ± standard deviation with mean values marked above the columns.

The values of the zeta potential are positive for all conjugates studied (Figure 4). The vehicle G3^2B12gh^ has a ζ of circa 11 mV, the same as the G3^16gh^ studied in a regular series of derivatives [39]. αM-containing conjugates showed the same ζ, circa 22 mV, which is twice that for the vehicle itself, despite the 10-fold size difference between G3^2B12gh5M^ and G3^2B10gh17M^ in water. On the other hand, the potential increased considerably upon a two log increase of hydrogen cation concentration, presumably due to protonation of core PAMAM and considerable increase of cationic charge of nanoparticles of the conjugates. For the size-dependent biological behavior of conjugates vide infra.

### 3.3. Cytotoxicity

The main goal of this study was to design an efficient αM delivery vehicle, based on the biotinylated and glucoheptoamidated (gh) PAMAM G3 dendrimer, increasing the drug’s toxicity against cancer cells and *C. elegans*. While substitution by biotin was supposed to increase the intracellular accumulation of the conjugates, modification by gh residues was expected to reduce the toxicity of the surface groups of the αM carrier (PAMAM G3 dendrimer).

**Figure 5:**
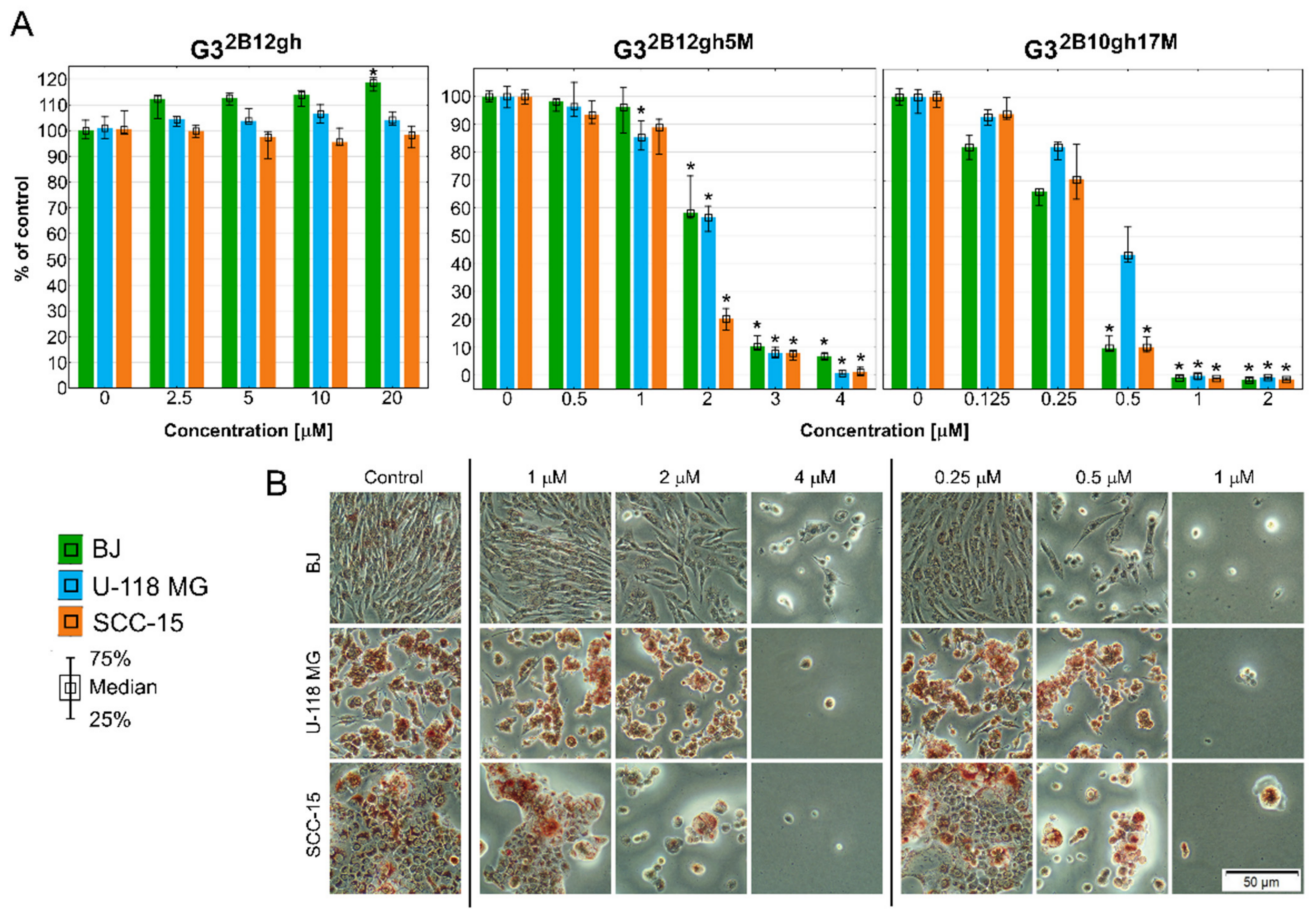
Cytotoxicity of G3^2B12gh5M^, G3^2B10gh17M^, and the control vehicle G3^2B12gh^ against human cells: normal fibroblasts (BJ), glioma cells (U-118 MG), and squamous carcinoma cells (SCC-15) after 48 h of incubation estimated with an NR assay. (**A**) Cell viability expressed as medians of a percent against non-treated control (control expressed as 100%). The whiskers are the lower (25%) and upper (75%) quartile ranges. * *p* ≤ 0.05; Kruskal–Wallis test (against non-treated control). (**B**) Morphology of the cells incubated with chosen concentrations of G3^2B12gh5M^ and G3^2B10gh17M^. Red signal on microscopic images represents a neutral red dye.

The results of the NR assay (Figure 5) revealed that both studied αM conjugates were active at low, micromolar concentrations, with the effect depending on the level of dendrimer substitution by the drug. Thus, G3^2B10gh17M^ killed all cells more efficiently than G3^2B12gh5M^. Surprisingly, normal cells were as sensitive as SCC-15 cells, with glioma cells being less sensitive. The results were confirmed by microscopic analysis. At toxic or higher concentrations, the studied compounds cells formed aggregates, shrank, lost their adhesion, and absorbed a lower amount of neutral red dye (Figure 5B).

**Figure 6:**
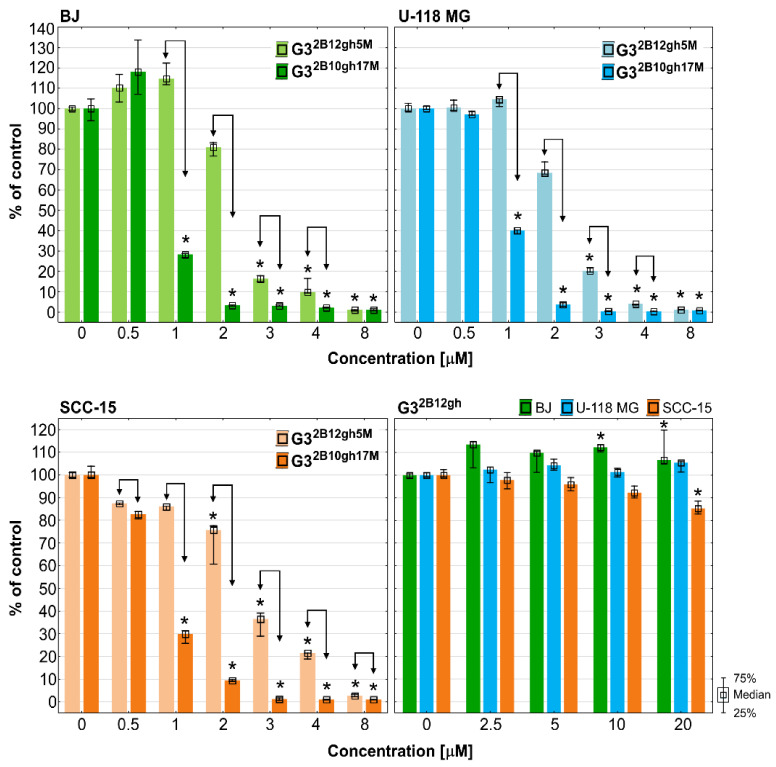
The results of XTT assay performed on BJ, U-118 MG and SCC-15 cells after 48 h of incubation with G3^2B12gh5M^ and G3^2B10gh17M^ conjugates and the control vehicle G3^2B12gh^. Cells viability expressed as medians of a percent against non-treated control (control expressed as 100%). The whiskers are lower (25%) and upper (75%) quartile ranges. * *p* ≤ 0.05; Kruskal–Wallis test (against non-treated control), ↕ *p* ≤ 0.05; Mann–Whitney *U* test (the G3^2B12gh5M^-treated group against the G3^2B10gh17M^-treated group).

The results of XTT assay, detecting mainly mitochondria damages, showed a weaker response than those of NR test (Figure 6). Of note is that despite the effect of the G3^2B12gh5M^ compound, compared to that of G3^2B10gh17M^ compound, the former seems to be a better candidate for the destruction of neoplastic cells, in view of a strong toxicity of the latter towards normal human fibroblasts (Figure 6, Table 2).

The results of the NR and XTT assays allowed to determine the half maximal inhibitory concentration IC_50_ values, demonstrating both drug conjugates to lack cytotoxic selectivity against BJ vs. squamous carcinoma SCC-15/glioma U-118 MG cells (Table 2, Figure 5 and Figure 6).

Of note is that with the control dendrimer vehicle G3^2B12gh^, void of αM, at concentrations up to 20 µM (the highest allowed in view of dmso in the dendrimer preparation) following 48 h of treatment, a feeble cytotoxicity of the highest concentration was seen only for SCC-15 cells with the XTT assay (Figure 5 and Figure 6). These results are in agreement with our earlier results, indicating that a similar G3 PAMAM dendrimer substituted with 16 gh residues was non-toxic up to 100 µM [39]. It confirms that biotinylated and partially glucoheptoamidated PAMAM G3 dendrimer is useful as a drug nanocarrier. The conjugation of αM with non-toxic dendrimeric carrier (G3^2B12gh^) resulted in the drug’s toxicity increase, as compared to that of the free αM [5]. The increase of toxicity was dependent on the level of substitution of the carrier by the drug, observed not only against SCC-15 cells (about 4.5- or 21-fold), but also for U-118 MG (about 5.2- or 25-fold) and BJ cells (4.5- or 11.5-fold for G3^2B12gh5M^ and G3^2B10gh17M^, respectively). It should be noted that the conjugate with five residues of αM enhanced its toxicity on average by ca. 5-fold, which may be related to the polyvalency or multivalency phenomenon [40]. Of interest is that the conjugate with 17 residues enhanced αM activity by about 21–25-fold in cancer cells but by only 11.5-fold in normal cells. This suggests that addition of more xanthone residues increase its activity in cancer cells with simultaneous moderation of this effect in normal cells, resulting in an apparent enhancing of antineoplastic efficacy [41].

It should also be mentioned that U-118 MG cells, compared to the remaining cell lines, did not present higher sensitivity to the drug bound to both biotinylated vehicles, which was unexpected considering that glioma cells tend to overexpress biotin receptors and in consequence demonstrate increased biotin uptake [42,43].

### 3.4. Cellular Accumulation and Distribution of Fluorescently Labeled G3^2B12gh5M^ or G3^2B10gh17M^

Drug vehicles, including dendrimers, may change cellular uptake and accumulation of delivered substances. Based on our earlier experience [22] and considering higher cellular uptake of biotinylated vs. non-biotinylated dendrimers [20,21] the glucoheptoamidated dendrimers used in this study were substituted with two biotin residues per dendrimer molecule, in order to enhance their accumulation and, in consequence, also αM accumulation in the studied cancer cells. It should be added that an increased uptake of biotinylated dendrimer-drug conjugates by cancer cells could be expected, in view of biotin receptors and transporters being frequently overexpressed by such cells [19].

**Figure 7:**
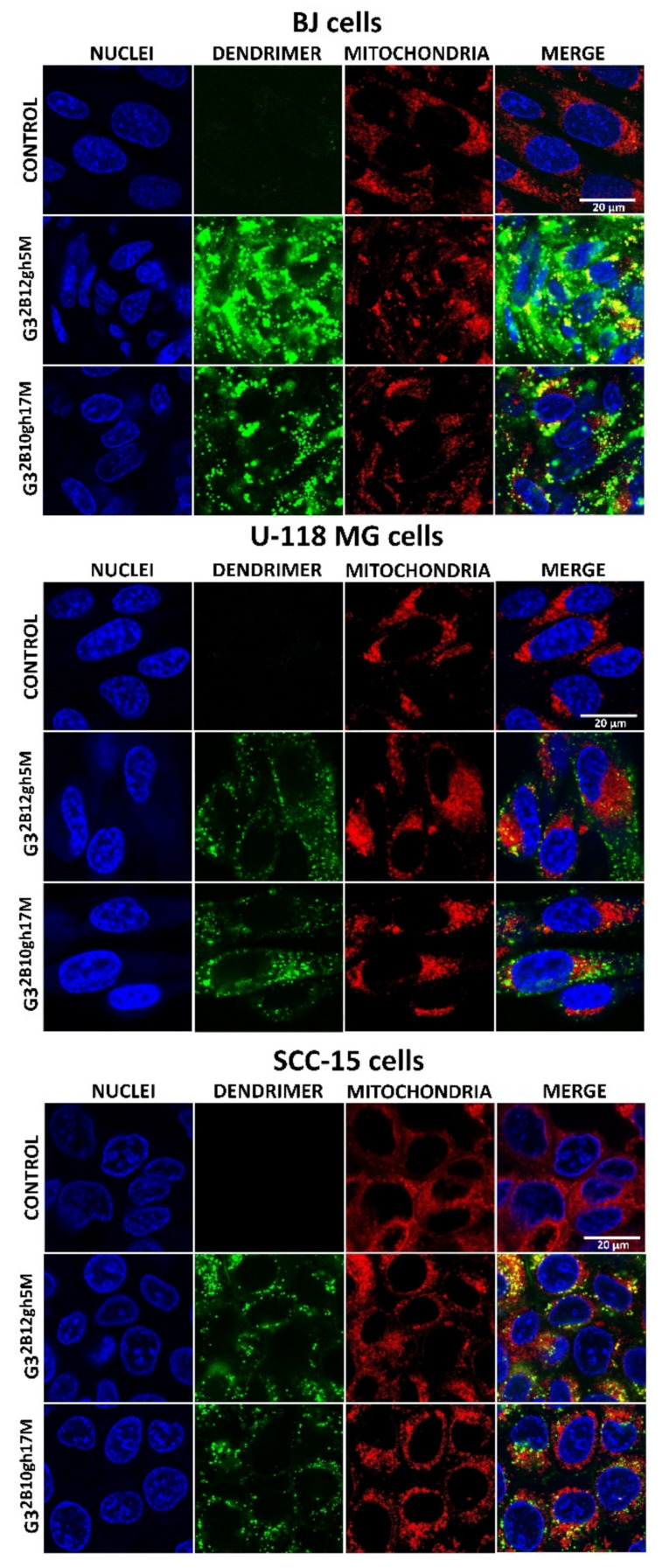
Images obtained with confocal microscopy presenting cellular accumulation and localization of fluorescently labeled G3^2B12gh5M^ or G3^2B10gh17M^ conjugates after 48 h of incubation with 1 µM or 0.1 µM non-toxic concentrations. Green signal: FCH-labeled dendrimer, blue signal: cell nuclei stained with Hoechst 33342, red signal: MitoTracker Deep Red FM labeled mitochondria, yellow signal: colocalization of FCH labeled dendrimer and MitoTracker labeled mitochondria. Scale bar is equal 20 μm.

Cellular uptake, accumulation and toxicity are dependent on molecule size, charge and charge-related zeta potential of nanoparticles. Increased cytotoxicity of nanoparticles in non-phagocytic cells correlates with their small size [44]. At physiological pH, G3^2B12gh5M^ and G3^2B10gh17M^ conjugates indicated high association with diameter determined by DLS, equal to about 1300 nm for G3^2B12gh5M^ and 130 nm for G3^2B10gh17M^ compared to an αM-free carrier (about 1 nm diameter). Probably for this reason, microscopic observations of cells after 4 h of incubation with both FCH-labeled G3^2B12gh5M^ and G3^2B10gh17M^ revealed no visible fluorescence signal inside them. Only 48 h of incubation of fluorescently labeled G3^2B12gh5M^ or G3^2B10gh17M^ indicated that both compounds were taken up by all cell lines with the highest accumulation in normal human fibroblasts, while in cancer cells, their amount was significantly lower (Figure 7). This phenomenon may be due to the ability of fibroblasts to more efficiently uptake biotin compared to other cells as we described [22]. It is also possible that the stronger accumulation of studied compound in these cells may be a result of their ability to non-professional phagocytosis or phagocytosis [45,46]. The formation of associates by αM conjugates resulted not only in slower uptake of these nanoparticles—the toxicity was also caused by the presence of αM itself rather than by the interaction of positively charged dendrimer vehicles and negatively charged cell membranes. Microscopic observations revealed that absorbed conjugates remained in endocytic vesicles, with only a small part of the dendrimer present in the cytoplasm of the cells. It is known that αM induces caspase-3 and -9 activation causes swelling, loss of membrane potential (delta psi), decrease in intracellular ATP, ROS accumulation, and cytochrome c/AIF release in human leukemia HL60 cells [47] and also provokes the release of cytochrome C, increase of Bax, decrease of Bcl-2, and activation of caspase-9/caspase-3 cascade in cervical cancer cells [33]. Therefore, mitochondria seem to be a natural goal for the anticancer activity of αM. In this study, both conjugates penetrated into mitochondria of SCC-15 cells and normal fibroblasts, but very poorly into mitochondria of glioma U-118 MG cells (Figure 7), suggesting a reason of glioblastoma cells being resistant to αM conjugates (see Figure 5 and Figure 6).

### 3.5. Caspase-3/7 and Intracellular ATP Level

**Figure 8:**
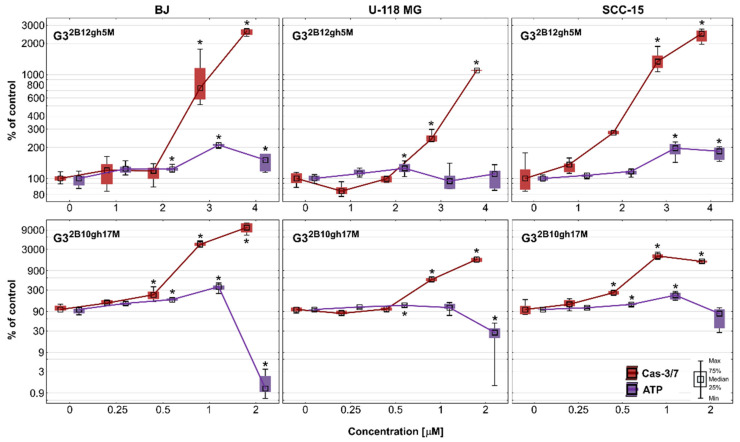
Activity of caspase-3/7 and intracellular ATP level in BJ, U-118 MG, and SCC-15 cells after 48 h of incubation with G3^2B12gh5M^ (1–4 μM) and G3^2B10gh17M^ (0.25–2 μM) conjugates. The results from three independent experiments are medians expressed as a percentage of non-treated control, where data for caspase-3/7 level are marked in red, while data for ATP level are marked in violet. Boxes represent the first (25%) and third (75%) quartile; whiskers are the minimum and maximum range. * *p* ≤ 0.05, Kruskal–Wallis test (against non-treated control).

Another sign of advanced cytotoxicity is the entry of cells into the programmed pathway of death, apoptosis. Regardless of whether this is an extrinsic or intrinsic mechanism, executive caspases (caspase 3,6,7) are produced to proteolytically degrade components of damaged or malfunctioning cells [48]. In this experiment, the potential of dendrimer conjugates with 5 or 17 residues of αM to induce apoptosis in cancer cells compared to normal cells was studied. In addition, the intracellular ATP level was also estimated. The results are shown in Figure 8 as a relationship between the activity of executioner caspases and intracellular ATP level in studied cell lines. Both conjugates G3^2B12gh5M^ and G3^2B10gh17M^ significantly increased activity of caspase-3/7 in all tested cell lines at concentrations higher than IC_50_. The most active was the conjugate with 17 residues of αM, inducing Cas-3/7 activity growth with a concentration of 0.5 μM, while the conjugate with 5 αM residues induced this effect with a concentration of 3 μM.

As the cytotoxicity of αM significantly increases upon the conjugation with the dendrimer, the same effect is observed for apoptosis. In our earlier studies [5], αM alone induced apoptosis from 10 μM in cancer and from 20 μM concentration in normal cells. Thus, lower concentrations of αM affected cancer than normal cells. Two other similar xanthones, namely alvaxanthone and rheediaxanthone B, induced higher activity of caspases-3/7 in cancer cell lines comparing to normal cells [49]. For αM conjugated with PAMAM G3 dendrimer, this effect was not seen—the most sensitive were fibroblasts. Referring to non-treated control, at the highest concentration of G3^2B12gh5M^, there was a 11-, 25- and 26-fold increase in caspases activity in U-118 MG, SCC-15, and BJ cells, respectively. In case of G3^2B10gh17M^, there was 17-fold growth in glioblastoma cells and 21-fold growth in the SCC-15 cell line, while fibroblasts obtained a 104-fold increase of caspases activity. This result may be due to the presence of biotin in conjugates and its higher uptake by fibroblasts compared to remaining cancer cells, as previously observed by Uram et al. [22]. The results of the accumulation studies of fluorescently labeled G3^2B12gh5M^ and G3^2B10gh17M^ conjugates are consistent with this, demonstrating higher uptake of biotinylated conjugates by fibroblasts (Figure 7). On the other hand, the lowest level of caspases activity in glioblastoma cells may be caused by their intrinsic mechanisms of decreased apoptosis, which makes GBM resistant to apoptosis inhibitors [50] and causes the propensity of astrocytic glioma to necrosis [51]. Considering this fact, the obtained statistically significant induction of apoptosis by αM dendrimer conjugates at low concentrations makes them interesting in the context of anticancer properties.

Since ATP plays a key role in the decision of cell death fate and the ATP-dependent nature of apoptosis [48,52], intracellular ATP level after 48 h of incubation with G3^2B12gh5M^ and G3^2B10gh17M^ was assessed. After treatment with 1–3 μM G3^2B12gh5M^, the ATP amount has grown to 195% in SCC-15 cells and to 210% in BJ cells, and then, at the highest concentration, this was 183% and 150%, respectively. In the case of U-118 MG cells, the level of ATP remained mostly unchanged. In the same range of concentrations, activity of caspases has grown significantly in all three cell lines. Meanwhile, incubation of both cancer and normal cells with G3^2B10gh17M^ led to an increase in ATP level at 1 μM, followed by a reduction at 2 μM concentration. The biggest rise and fall of ATP level was observed in fibroblasts (363% and 1.2% of control), while in SCC-15 cells it was 225% and 80%. In glioblastoma cells, the ATP level increased only to 112%, but then decreased to 27%. These ATP changes were accompanied by growth in caspases-3/7 activity in all tested cells, alike for G3^2B12gh5M^. As a high level of intracellular ATP often favors apoptosis [52], and caspases 3 and 7 are activated in the advanced stage of this process, it can be concluded that the studied conjugates induced ATP-dependent apoptosis in a concentration-dependent manner. However, considering the fact that various forms of cell death (autophagy, apoptosis, and necrosis) can be induced simultaneously or successively and that low level of ATP often promotes necrosis [52], the tested conjugates at highly toxic concentrations (4 μM of G3^2B12gh5M^ and 2 μM of G3^2B10gh17M^) could also induce necrosis in both cancer and normal cells. A similar effect was observed by Uram et al. [53] in studies regarding biotinylated dendrimer PAMAM G3 conjugated with celecoxib and Fmoc-L-Leucine (G3-BCL). In fibroblasts, the apoptotic pathway was dominant up to 2 μM of G3-BCL conjugate with a constant ATP supply, while at 4 μM concentration, a significant increase of late apoptosis/necrosis was observed with simultaneous extensive depletion of ATP level. The same pattern of cell death fate was revealed in glioblastoma cells but still with a high level of ATP observed at 4 μM of G3-BCL.

### 3.6. Proliferation

**Figure 9:**
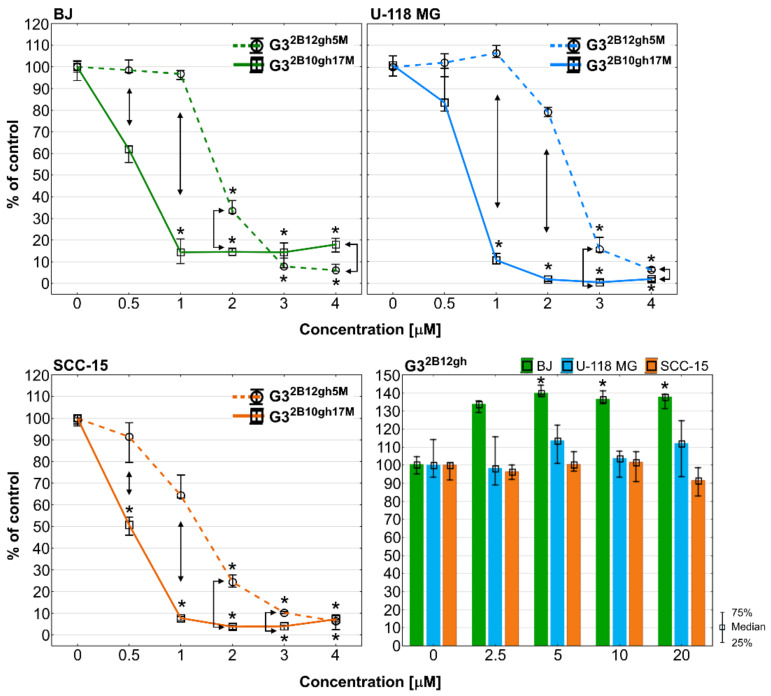
Anti-proliferative action of G3^2B12gh5M^ and G3^2B10gh17M^ on BJ, U-118 MG, and SCC-15 cells after 72 h of incubation, determined with Hoechst staining. Results are from three independent experiments, performed in triplicates and presented as medians (percentage of non-treated control). Whiskers indicate the lower (25%) and upper (75%) quartile ranges. * *p* ≤ 0.05, Kruskal–Wallis test (against non-treated control). ↕ *p* ≤ 0.05; Mann–Whitney *U* test (the G3^2B12gh5M^-treated group against the G3^2B10gh17M^-treated group).

It is known that αM exhibits a strong anti-proliferative effect in human colon HCT116 carcinoma cells with inhibition of the activity of DNA topoisomerases I and II, and blockade of the cell cycle in the G2/M phase [54], and also in human breast cancer T47D cells [55] and several others [56]. G3^2B12gh5M^ inhibited BJ and SCC-15 cell proliferation from 2 µM concentration and U-118 MG glioma cells from 3 µM, while at higher concentrations cell proliferation was lower than 20% and close to zero. A stronger effect was observed in case of G3^2B10gh17M^, which significantly suppressed proliferation from 1 µM concentration for BJ and U-118 MG or even 0.5 µM for SCC-15 cells. Moreover, activity of conjugate with 17 αM residues was always stronger than G3^2B12gh5M^. It is worth noting that the control dendrimer vehicle G3^2B12gh^ at concentrations up to 20 µM showed no anti-proliferative effect against tested cell lines and even increased the proliferation of fibroblasts by 40% (Figure 9).

Comparing this effect with the toxicity results, it can be concluded that the anti-proliferative effect coincides with the toxicity pattern (Figure 5 and Figure 6). It is also visible that conjugation of αM with PAMAM G3 dendrimers enhanced its antiproliferative effect since our previous studies showed activity of αM alone against BJ, U-118 MG and SCC-15 cells in range of 7.5–20 µM concentrations [5]. Finally, the use of the biotinylated carrier for 5 αM residues led to a reduction of cell proliferation about three-fold, while G3^2B10gh17M^ inhibited cell divisions by 7-, 15-, and 10-fold in BJ, U-118 MG, and SCC-15 cells, respectively, compared to αM alone. This phenomenon may be the result of multi/polyvalency effect or higher amount of αM accumulation inside the studied cells and showed that the studied compounds may play an important role as new anticancer agents.

### 3.7. Adhesion

**Figure 10:**
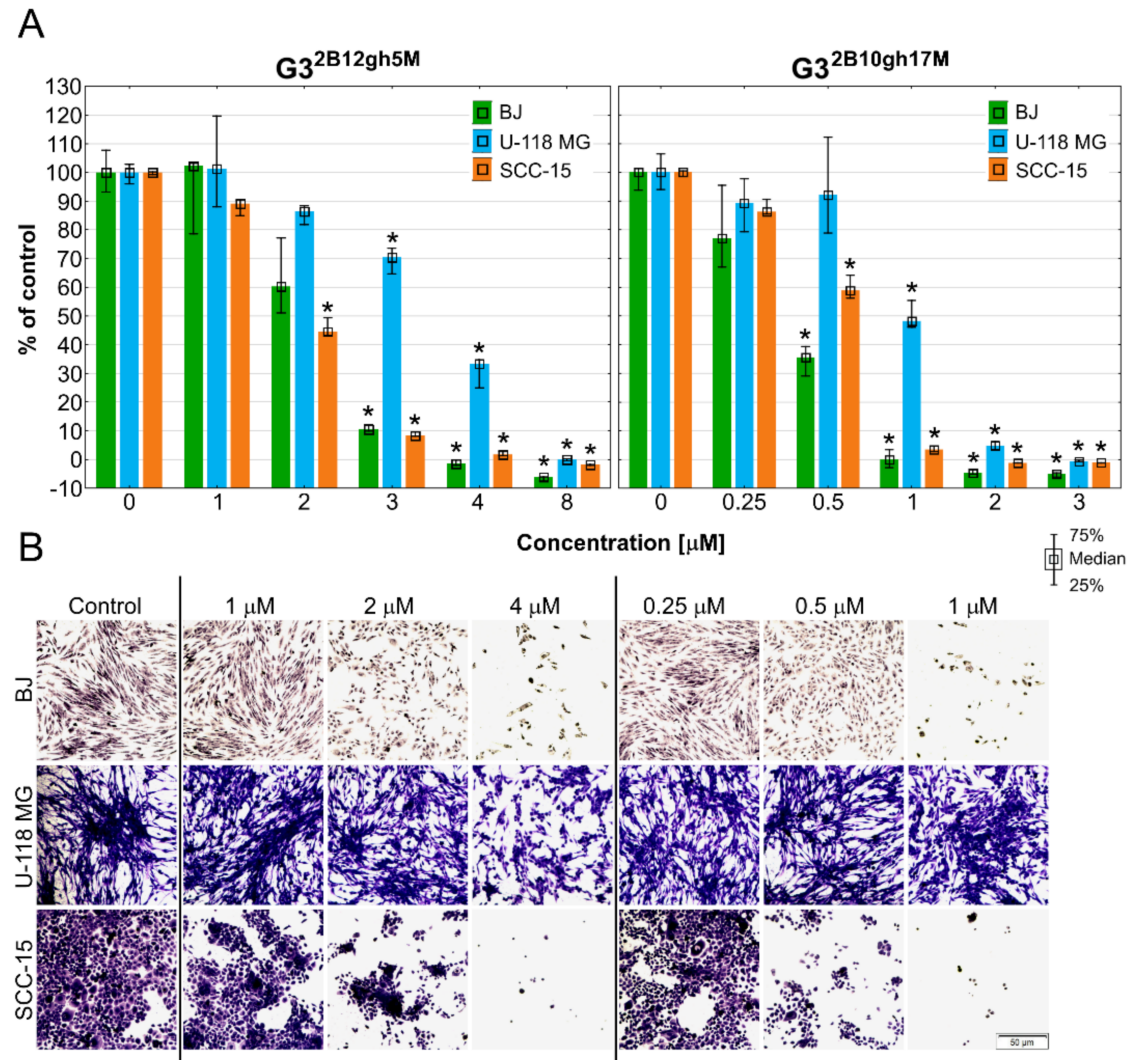
The ability of BJ, U-118 MG, and SCC-15 cells to adhere determined by crystal violet assay after 48 h of treatment with G3^2B12gh5M^ or G3^2B10gh17M^ at different concentrations. (**A**) Results are medians with first (25%) and third (75%) quartile obtained in three independent experiments and expressed as a percentage of non-treated control. * *p* ≤ 0.05, Kruskal-Wallis test (against control group). (**B**) Cells attached to the bottom of 96-well plate and stained with crystal violet after 48 h of incubation with studied dendrimer conjugates. Violet signal represents a crystal violet dye inside the cells. Scale bar = 50 μm.

Among the ten hallmarks of cancer development and progression, there is the activation of invasion and metastatic processes [57]. Malignant cells during migration adhere to extracellular matrix (ECM) proteins, providing a path to the new metastasis site [58,59]. As cell-to-cell and cell-to-ECM adhesion plays a very important role in tumor metastasis, the molecules with anti-adhesion properties may contribute to suppress the cancer expansion to other tissues. The ability of αM dendrimer conjugates to affect cells adhesion was evaluated by the assay with crystal violet dye, which stains DNA of intact adherent cells. The obtained results are presented in Figure 10.

The SCC-15 cells were the most susceptible to loss of adhesion after incubation with G3^2B12gh5M^, having 45% of attached cells at 2 μM and 1.7% at 4 μM concentration. Fibroblasts and glioblastoma cells achieved a statistically significant decrease of adhesion at 3 μM of G3^2B12gh5M^, but the difference between these two cell lines was 60% in favor of U-118 MG cells. PAMAM G3 dendrimer with 17 residues of αM influenced cells attachment stronger, significantly decreasing adhesion of BJ and SCC-15 cells from 0.5 μM concentration. For this conjugate, U-118 MG cells were also the least affected, having 48% of adherent cells at 1 μM, which is 12 times more than for BJ and SCC-15 cells. These results are in line with the microscopic observations shown in the Figure 10B, which present the number of adherent cells stained with crystal violet dye after incubation with tested conjugates in selected concentrations. U-118 MG cells retained relatively high adhesiveness even after treatment with conjugates at concentrations higher than the IC_50_ values. Similar results were obtained for αM and other xanthones [5,49]. In these studies, the adhesion of squamous carcinoma cells was slightly more disturbed compared to fibroblasts, while the weakest effect was observed in glioblastoma cells. Considering the cytotoxicity of the tested conjugates, the G3^2B12gh5M^ conjugate seems to be more potent in anti-adhesion properties in squamous cell carcinoma treatment. In case of GBM, extracranial metastases are extremely rare, affecting 0.4–0.5% of all patients with GBM [60], but in the last decade, clinical reported cases of extraneural dissemination have become more frequent [61]. In addition, GBM is suggested to disseminate not only via cerebrospinal fluid, but also through bloodstream and lymphatic vessels. Therefore, the ability of G3^2B10gh17M^ to reduce adhesion of U-118 MG cells by 52% at 1 μM concentration is noteworthy.

### 3.8. Toxicity to C. elegans and the Effect on the Worm Survival

**Figure 11:**
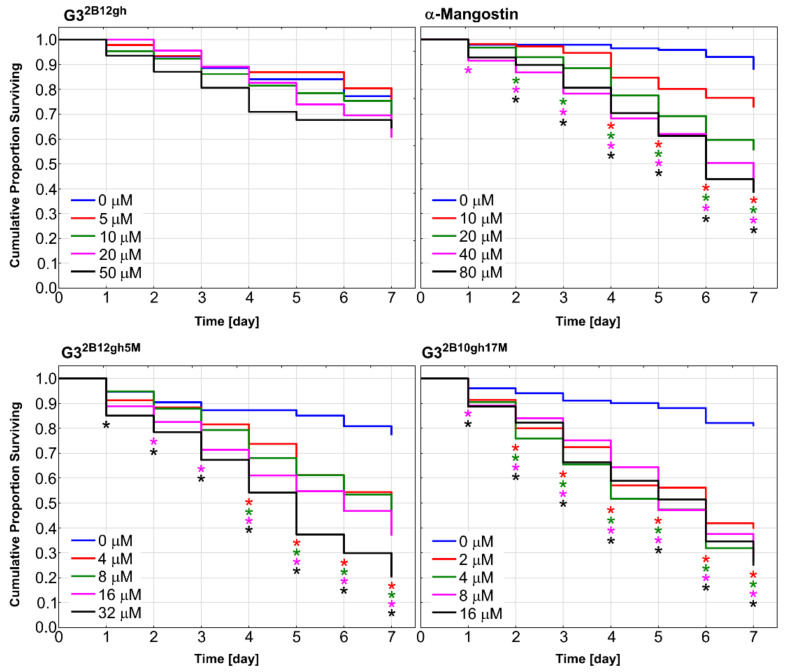
Kaplan–Meier survival curves of *C. elegans* after 7 days of incubation with α-mangostin, G3^2B12gh5M^, G3^2B10gh17M^, and G3^2B12gh^ vehicle at different concentrations. Results are presented as cumulative proportion surviving. Statistically significant differences against non-treated control obtained in Gehan’s Wilcoxon test are marked with asterisks (*p* ≤ 0.05) in the colors corresponding to the tested concentrations.

*Caenorhabditis elegans* nematode was used as a model organism to examine in vivo effect of synthesized αM dendrimer conjugates on multicellular system. It has been shown that *C. elegans* is valuable animal model for studies on toxicity and biocompatibility of various nanoparticles [62,63]. Synchronized population of L4 stage-worms was incubated for 7 days in the medium (see Section 2.5.7) containing different concentrations of αM, G3^2B12gh5M^ and G3^2B10gh17M^, as well as G3^2B12gh^ (control vehicle). The results are presented in Figure 11 as survival curves determined by the Kaplan–Meier method.

Similar to the results of the in vitro studies, attachment of αM to dendrimer vehicle increased its toxicity against *C. elegans* organism, but to a slightly lesser extent. The concentrations in which the proportion of live nematodes was 0.4 after 7 days of incubation were 40 μM of αM, 16 μM of G3^2B12gh5M^, and 2 μM of G3^2B10gh17M^ (Figure 11), pointing to the activity of G3^2B12gh5M^ and G3^2B10gh17M^, compared to free αM, being 2.5- and 20-fold stronger, respectively.

**Figure 12:**
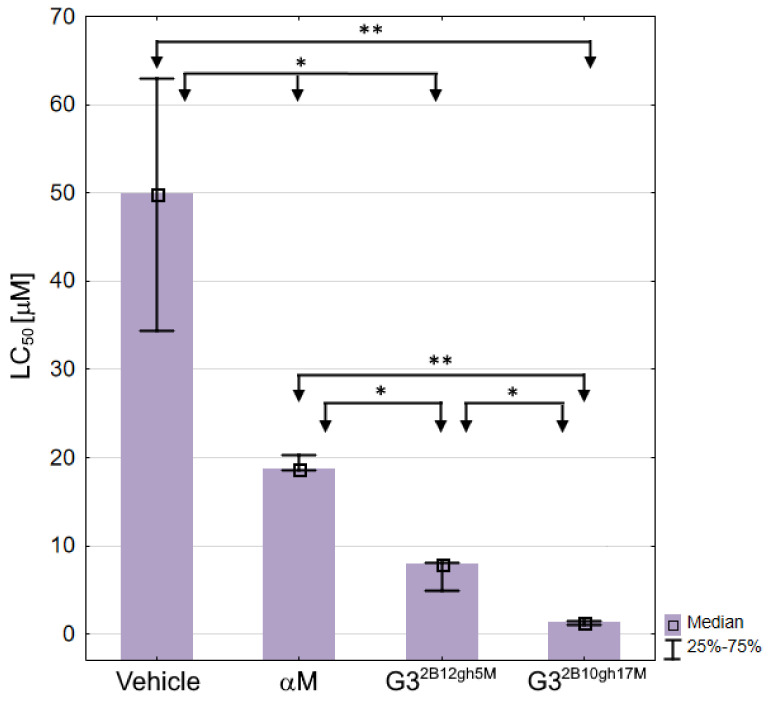
The LC_50_ values against *C. elegans* for αM, dendrimer vehicle G3^2B12gh^ and G3^2B12gh5M^ or G3^2B10gh17M^ conjugates presented as medians with the first and third quartile. Statistically significant differences estimated by the Kruskal–Wallis test are marked with ** and by U Mann–Whitney test with * *p* ≤ 0.05.

Based on the survival curves after 7 days of incubation, the LC_50_ values were also calculated for αM, G3^2B12gh5M^, G3^2B10gh17M^, and control vehicle G3^2B12gh^ (Table 3, Figure 12), the latter showing a moderate toxicity level [64].

As expected, each of the G3^2B12gh5M^ and G3^2B10gh17M^ conjugates was more toxic than the G3^2B12gh^ control vehicle (by 6.3- and 36-fold, respectively) or free αM, with the activities of G3^2B12gh5M^ and G3^2B10gh17M^, compared to free αM, being stronger by 2.4- and 13.6-fold, respectively (Table 3).

The present results obtained for free αM, tested against the L4 stage nematodes (Table 3), differ from those obtained earlier in toxicity assay performed by a different method and against a mixed *C. elegans* population [5]. The LC_50_ value for αM was found 3.8 ± 0.5 μM, thus the nematode at L4 stage (LC_50_ = 18.74) appears distinctly less sensitive to the drug than the other developmental forms.

**Figure 13:**
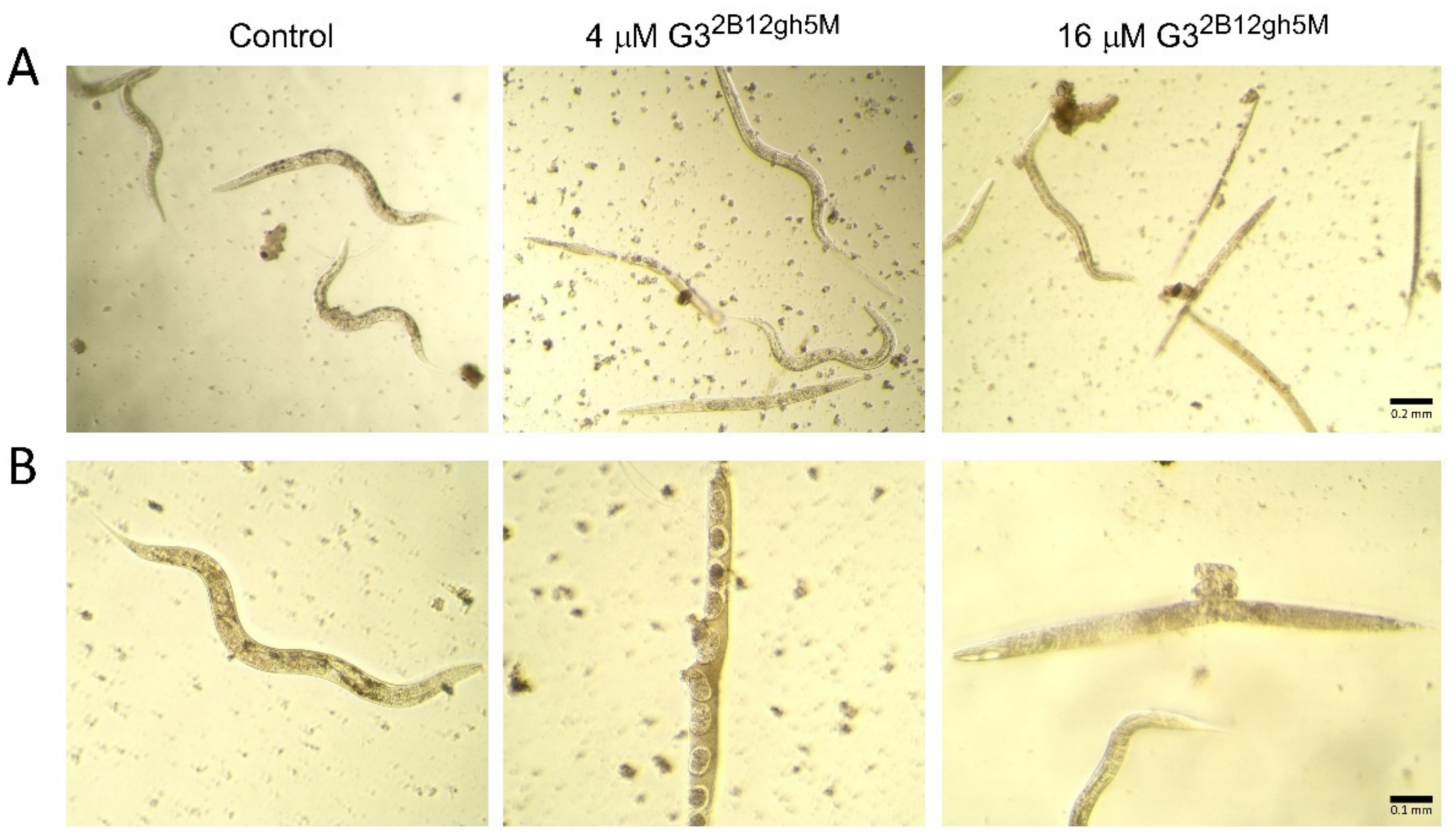
Morphology of *C. elegans* nematodes after 7 days of incubation with G3^2B12gh5M^ dendrimer at a concentration of 4 and 16 μM in comparison to the control group representative, presented under 10× (**A**) and 20× (**B**) objective magnification.

The toxic effects of αM and its conjugates with biotinylated PAMAM G3 dendrimer was accompanied by morphological and locomotion changes of the treated nematodes. Already after 24 h of incubation with the highest concentrations of G3^2B12gh5M^ and G3^2B10gh17M^, slower and vibrating movements were observed. As the toxic effect progressed, the wavy motion declined, leading to straightening and stiffening of the *C. elegans* worm, until it became motionless. Moreover, the creases of the cuticula and degradation of this tissue were seen (Figure 13).

It should be noted that the increase of αM toxicity, resulting from the drug binding with the dendrimer molecules in G3^2B12gh5M^ and further enhancement in consequence of the higher level of dendrimer substitution by αM in G3^2B10gh17M^, was similar when tested in vivo with *C. elegans* (by 14-fold in terms of the LC_50_ values; Table 3) and in vitro with cells (by 9–32-fold considering the IC_50_ values; Table 2). Thus, the free drug and its dendrimer-bound forms appear to interact with cells and the model organism following a common mechanism. Further studies are needed to follow the dendrimer-bound αM uptake by *C.elegans* and compare it with that documented for certain other nanomolecules [65,66,67], such a study being impeded by a certain level of the worm autofluorescence.

## 4. Conclusions

Since α-mangostin is considered a useful agent in anticancer therapy, it seems necessary to try to increase its solubility, efficacy and selectivity by finding a suitable carrier for it. In this study, we showed that non-toxic, third-generation glucoheptoamidated and biotinylated poly(amidoamine) dendrimers can provide an excellent carrier for αM, making its cytotoxic, antiproliferative, and anti-adhesive properties more visible at significantly lower concentrations. Additionally, it gives the opportunity to avoid side effects associated with a too high concentration of αM in therapy. At the same time, the mechanism of action of the described conjugates remains characteristic for αM itself (pro-apoptotic effect and changes in ATP levels related to the mitochondrial pathway). This means that despite the lack of selectivity against neoplastic cells, the studied conjugates may be a valuable tool in the local therapy of glioblastoma and squamous cell carcinoma. It is worth to underline that the dendrimer vehicle at concentrations up to 20 µM showed no anti-proliferative effect against tested cell lines, with a feeble cytotoxicity of the highest concentration seen only with squamous carcinoma cells. The present results indicate that the proposed αM delivery system allows the drug to be more effective in the dendrimer-bound than in the free state against both cultured cancer cells and the model organism, suggesting that this treatment is promising for anticancer as well as anti-nematode chemotherapy.

## Figures and Tables

**Table 1:** Size and zeta potential values ± standard deviation determined by DLS analysis.

Compound	Size [nm]				Zeta Potential [mV]	
	pH 7		pH 5		pH 7	pH 5
	d(V)	d(N)	d(V)	d(N)		
G3^2B12gh^	1.0 ± 0.24	0.9 ± 0.22	4.5 ± 0.14	3.8 ± 0.18	11.1 ± 2.89	17.4 ± 1.87
G3^2B12gh5M^ G3^2B10gh17M^	1367 ± 245.7	1262 ± 196.4 111 ± 14.1	178.3 ± 5.73 230.6 ± 12.4	113.8 ± 7.34	22.7 ± 1.01	37.5 ± 2.32 39.5 ± 3.67
	149 ± 33.9			130.8 ± 12.25	22.1 ± 0.61	

**Table 2:** The half maximal inhibitory concentration (IC_50_) values determined following 48 h of treatment of BJ, U-118 MG, or SCC-15 cells with G3^2B12gh5M^ or G3^2B10gh17M^.

IC_50_ [μM] NR Assay			
	BJ	U-118 MG	SCC-15
G3^2B12gh5M^	2	1.83	1.41
G3^2B10gh17M^	0.28	0.39	0.31
α-Mangostin^1^	8.97	9.59	6.43
**IC_50_ [μM] XTT Assay**			
	BJ	U-118 MG	SCC-15
G3^2B12gh5M^	2.37	2.05	2.52
G3^2B10gh17M^	0.78	1.01	0.85
α-Mangostin ^1^	18.58	18.15	7.72

^1^ IC_50_ values for α-mangostin quoted from our earlier work [5].

**Table 3:** The LC_50_ values against *C. elegans* for free and dendrimer-bound αM, and a control dendrimer vehicle, determined following 7 days of incubation with αM, G3^2B12gh5M^, G3^2B10gh17M^, or G3^2B12gh^ presented as medians and the first and third quartile. The LC_50_ values were determined using an online calculator (AAT Bioquest, Inc. Quest Graph™ IC_50_ Calculator. Retrieved from https://www.aatbio.com/tools/ic50-calculator (accessed on 14 October 2021).

	LC50 [μM]	1st Quartile	3rd Quartile
G3^2B12gh^	49.88	40.58	57.98
α-Mangostin	18.74	18.64	20.23
G3^2B12gh5M^	7.87	4.95	8.09
G3^2B10gh17M^	1.38	1.03	1.42

## Data Availability

Data supporting the reported results are available on request from the corresponding author.

## Supplementary Materials

The following are available online at https://www.mdpi.com/article/10.3390/ijms222312925/s1.
