# Supplementary material for: Biotin Transport-Targeting Polysaccharide-Modified PAMAM G3 Dendrimer as System Delivering α-Mangostin into Cancer Cells and C. elegans Worms
Source: Int J Mol Sci. 2021 Nov 29;22(23):12925. doi: 10.3390/ijms222312925 (PMC8657743; doi:10.3390/ijms222312925)
Supplement: Supplementary file 1 [file ijms-22-12925-s001.zip › ijms-1456323-supplementary.pdf]

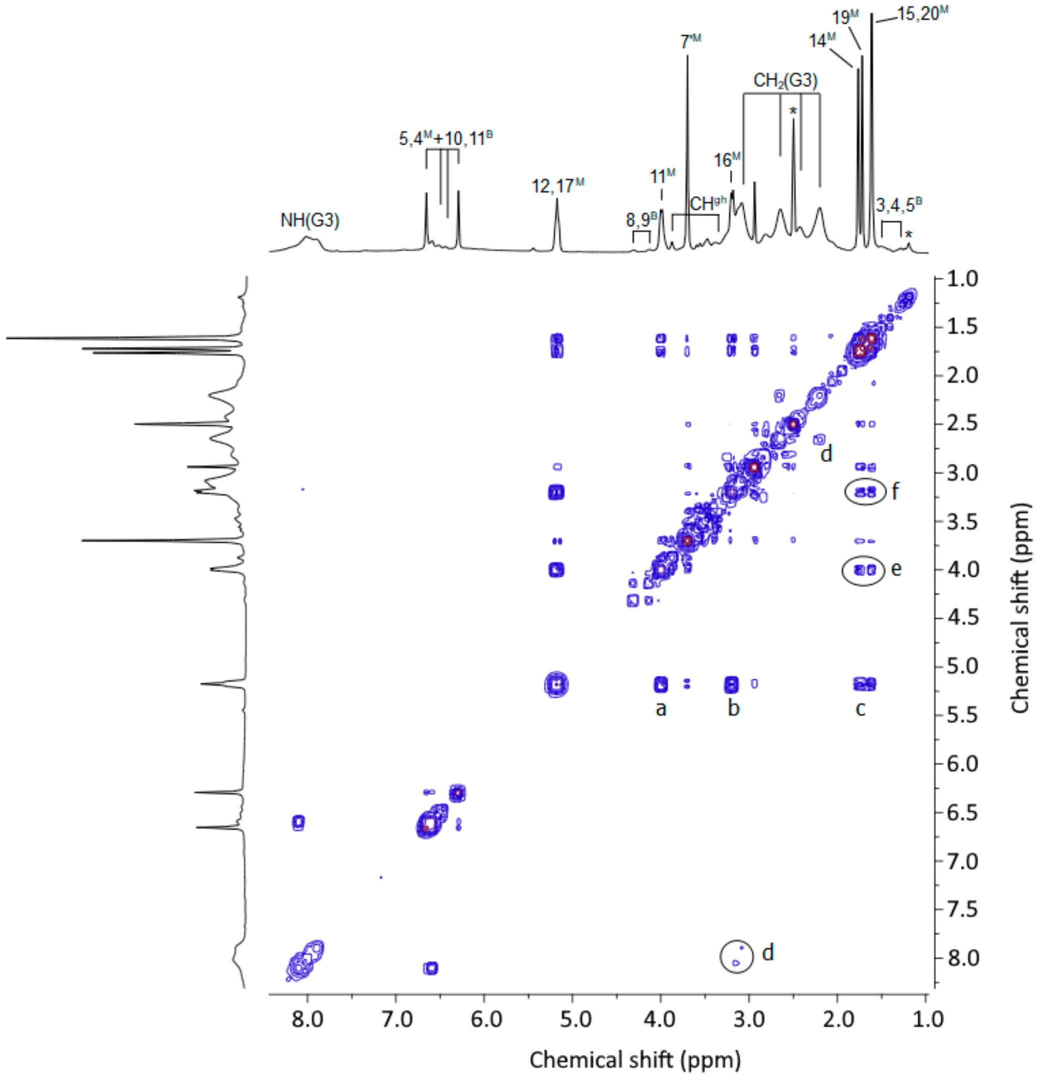

Figure S1. COSY spectrum for $G3^{2B10gh17M}$ in dmso-d_6. The relevant cross-peaks are labeled as follow: a - 12H/11H; b - 17H/16H; c – group of scalar coupling peaks between 14H, 15H, 19H, 20H (methyl group singlets) with 12H, 17H (overlapped methylene triplets); e - 14H,15H/11H; f - 19H,20H/16H; d – group of internal PAMAM G3 cross-peaks (not specified; for detailed assignment see [39].

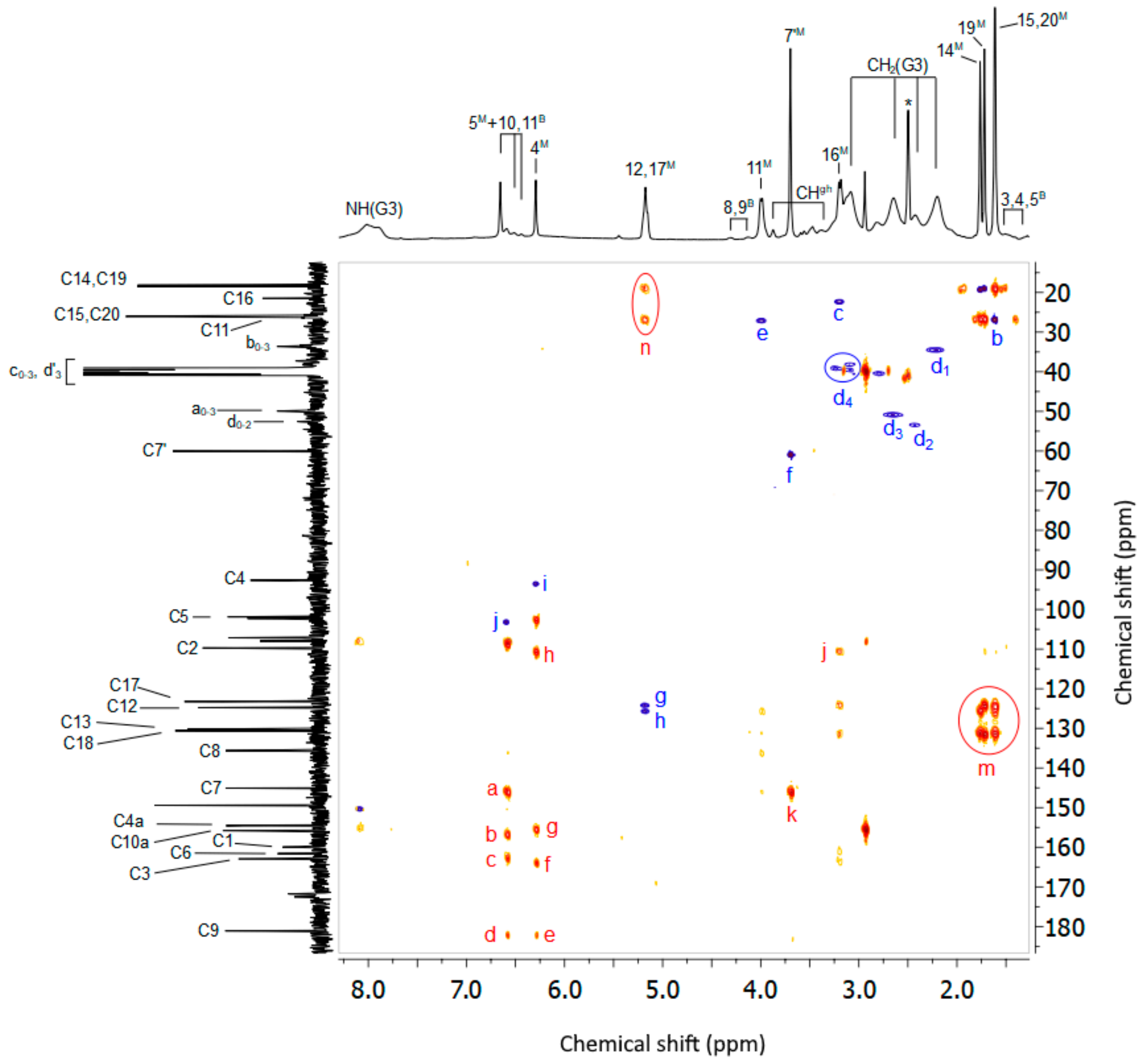

Figure S2. Combined HSQC/HMBC map for $G3^{2B10gh17M}$ in dmso-d_6. One-bond correlation peaks are shown and labeled in blue, while longer distance ^1H-^{13}C cross-peaks obtained in HMBC experiment are drawn and labeled in red-yellow scale. The following unambiguous HSQC peaks for internal G3 are: d_1 – $b_{0\text{-}3}$; d_2 – $d_{0\text{-}2}$; d_3 – $a_{0\text{-}3}$; d_4 – $c_{0\text{-}3}$,$d_{3'}$, while series of M residues HMBC are: a – H14,H19/C14,C19; b – H15,H20/C15,C20; c – H16/C16; e – H11/C11; f – H7′/C7′; g,h – H12,H17/C12,C17; i – H4/C4; ; j – H5/C5. The relevant HMBC peaks are as follows: a – H5/C7; b – H5/C10a; c – H5/C6; d – H5/C9; e – H4/C9; f – H4/C3; g – H4/C4a; h – H4/C2, j – H16/C2; k – H7′/C7, m – H14,H15/C12,C13 and H19,H20/C17,C18; n – H12,H17/C14,C15,C19,C20.

Table S1. The ^{1}H and ^{13}C resonance assignments for α-mangostin in G3^{2B10gh17M}

Locant (Scheme 1)	Chemical shift	
	^{13}C	^{1}H
1	160.0	-
2	109.7	-
3	162.9	-
4	92.6	6.29 [1H], s
4a	154.5	-
9a	154.9	-
9	181.2	-
8a	108.0	-
10a	155.8	-
5	101.9	6.65 [1H], s
6	161.6	-
7	145.1	-
7′	60.2	3.69 [3H], s
8	135.6	-
11	26.2	3.99 [2H], d
12	124.8	5.17 [1H], t
13	130.2	-
14	18.4	1.75 [3H], s
15	26.1	1.61[3H], s
16	21.6	3.19
17	123.3	5.17 [1H], t
18	130.6	-
19	18.2	1.72 [3H], s
20	26.0	1.61[3H], s
